# Probing beyond: The impact of model size and prior informativeness on Bayesian SEM fit indices

**DOI:** 10.3758/s13428-025-02609-2

**Published:** 2025-03-05

**Authors:** Ejike Edeh, Xinya Liang, Chunhua Cao

**Affiliations:** 1 Department of Counseling, Leadership, and Research Methods, University of Arkansas, 751 W. Maple St, Fayetteville, AR 72701 USA; 2 Educational Studies, College of Education, University of Alabama, 316A Carmichael Hall, Tuscaloosa, AL USA

**Keywords:** Bayesian factor analysis, Bayesian priors, Model size, Bayesian fit indices

## Abstract

Examining how well a hypothesized model fits observed data is pivotal in Bayesian structural equation modeling (SEM). Recent efforts have aimed to formulate Bayesian SEM fit indices akin to those used in frequentist SEM. However, the assessment of these fit indices in Bayesian SEM has been limited in scope. This research entailed two simulation studies to explore the impact of various factors, including prior choices, model misspecification, model size, and sample size, on the sensitivity of fit indices. The first study delved into the prior sensitivity of Bayesian SEM fit indices (BRMSEA [Bayesian root mean square error of approximation], BCFI [Bayesian comparative fit index], BTLI [Bayesian Tucker–Lewis index], and PP*p* [posterior predictive *p*-value]) under latent factor and cross-loading misspecifications. The second study explored how alterations in model complexity influenced the prior sensitivity of these fit indices to model misspecifications. The findings indicate a robust performance of Bayesian fit indices under relatively severe model misspecification, suggesting a preference for highly informative priors when prior knowledge is certain. As model size increased, PP*p* exhibited a balanced performance between true and false positive rates, whereas BRMSEA was less reliable in the presence of latent factor misspecification. Implications for applied research are discussed.

**Supplementary Information:**

The online version contains supplementary material available at 10.3758/s13428-025-02609-2.

Confirmatory factor analysis (CFA), as a statistical technique within the structural equation modeling (SEM) framework, is an important tool for modeling the relationship between items and latent factors. CFA models are commonly evaluated under the frequentist framework utilizing maximum likelihood estimation. Over the past decade, the Bayesian approach to SEM (Bayesian SEM or BSEM; Muthén & Asparouhov, 2012; Song & Lee, 2012) has emerged as a viable alternative to the frequentist approach. BSEM possesses inherent advantages, including the ability to integrate prior knowledge into analysis and improved performance with small sample sizes given appropriate priors (Kaplan & Depaoli, 2012; Muthén & Asparouhov, 2012; Van de Schoot et al., 2014). Despite the several advantages of the Bayesian approach, the evaluation of model fit in the context of BSEM remains an emerging research area.

Understanding how well a hypothesized model fits observed data is central to BSEM. Posterior predictive *p*-value (PP*p*) has been used to evaluate BSEM model fit from the onset. Although PP*p* has been shown to be appropriate for assessing overall model fit within small samples (Asparouhov & Muthén, 2010; Lee & Song, 2004; Rupp et al., 2004), it tends to be inconsistent when an informative (small variance) prior is specified (Hoijtink & Van de Schoot, 2018). PP*p* also increasingly signals model misfit for trivial differences between the observed data and hypothesized models as sample size increases (Asparouhov & Muthén, 2010).

There have been efforts in recent years to develop global BSEM fit indices similar to the approximate fit indices in frequentist SEM. Hoofs et al. (2018) first proposed Bayesian root mean square error of approximation (BRMSEA), analogous to frequentist-based RMSEA, for assessing the fit of BSEM models with large samples. This was closely followed by Garnier-Villarreal and Jorgensen (2020), who extended this idea beyond BRMSEA and developed the Bayesian comparative fit index (BCFI), Bayesian Tucker–Lewis index (BTLI), Bayesian normed fit index (BNFI), Bayesian gamma-hat (B $\widehat{\Gamma }$), and Bayesian McDonald’s centrality index (BM_C_).

The assessment of these BSEM global fit indices has been limited in scope, with only a few studies conducted (Asparouhov & Muthén, 2021; Edwards & Konold, 2022; Garnier-Villarreal & Jorgensen, 2020; Hoofs et al., 2018; Konold & Sanders, 2023; Liang, 2020; Winter & Depaoli, 2022a; Winter & Depaoli, 2022b). It is essential that the prior sensitivity of the recently introduced BSEM fit indices, including BRMSEA, BCFI, and BTLI, undergo a comprehensive investigation. Our study aims to systematically evaluate the sensitivity of BSEM fit indices across diverse sample sizes, levels of prior informativeness and accuracy, and with varying degrees of misspecification and model complexity.

The entire paper is organized as follows: We start with an introduction to the Bayesian CFA (BCFA) framework, prior specifications, key features of the recently developed BSEM fit indices, and a literature review. We then present the study simulation designs, results, discussions, and implications for applied research.

## Bayesian CFA framework

Generally, confirmatory factor analysis (CFA) can be specified as follows:

1 $$\mathbf{x}=\uptau +\Lambda \xi +\updelta$$

where **x** is a *p* × 1 vector of observed item responses, τ is a *p* × 1 vector of item intercepts, Λ is a *p* × *q* matrix of factor loadings, ξ is a *q* × 1 vector of latent factors, and δ is a *p* × 1 vector of measurement errors. It is assumed that δ is normally distributed and uncorrelated with ξ such that E(δ)=0 and cov(ξ, δ)=0. The Bayesian approach considers the uncertainty in all parameter estimates and hence treats them as random variables with a distribution. Bayesian estimation typically utilizes a Markov chain Monte Carlo (MCMC) algorithm, such as Metropolis–Hastings, Gibbs sampler (Geman & Geman, 1984; Hastings, 1970; Metropolis et al., 1953), or Hamiltonian Monte Carlo (Betancourt & Girolami, 2015; Neal, 2011), to iteratively generate a large sample of posterior distributions of target parameters $P\left(\theta |X\right)$, derived as a function of the data likelihood $P\left(\left.X\right|\theta \right)$ and prior knowledge $P(\theta )$, defined as

2 $$P\left(\theta |X\right)\propto P\left(X|\theta \right) P\left(\theta \right).$$

Specifically, given a set of parameters ξ, κ ,Φ ,τ , Λ and Ψ, the BCFA framework can be expressed as

3 $$P\left(\xi ,\kappa ,\Phi ,\tau ,\Lambda ,\Psi \left|\text{X}\right.\right)\propto P\left(X\left|\xi ,\kappa ,\Phi ,\tau ,\Lambda ,\Psi \right.\right) P\left(\xi ,\kappa ,\Phi ,\tau ,\Lambda ,\Psi \right)$$

where κ= E(ξ) is a mean vector, Φ is the covariance matrix of the latent factors, Ψ is the covariance matrix of the residuals, and all else as previously defined (Levy & Mislevy, 2016).

## Bayesian priors

The ability to incorporate prior knowledge into the modeling process is a fundamental advantage of the Bayesian approach. A prior is the probability distribution assigned to model parameters that denotes the initial beliefs about the values of the parameters from previous knowledge, research, or expert opinion (Gelman et al., 2013). The choice of priors depends on specific context and modelling assumptions, and is therefore inherently subjective. Even when the functional form of the prior type is known, assigning specific hyperparameters often poses a challenge, hence the use of diffuse or noninformative priors as the default prior in SEM software packages, including IBM SPSS Amos 21 (Arbuckle, 2012), blavaan in R (Merkle & Rosseel, 2018), and M*plus* (Muthén & Muthén, 1998–2023).

### The choice of a prior

Most prior choices are determined by two factors: the level of accuracy and the level of certainty of prior knowledge about the target parameter. A prior is considered accurate when the mean (median or mode) is centered on the target parameter value, and inaccurate otherwise (Depaoli, 2012; Finch & Miller, 2019). Regarding the level of certainty, informative priors (implying strong prior belief) are usually specified with small variances,^1^ while noninformative priors (indicating little or no prior knowledge) are specified with large variances. The greater the informativeness of a specified prior (i.e., the smaller the variance), the greater the impact on the realized posterior distribution of the target parameter, and vice versa.

The literature employs various terms to describe prior accuracy and informativeness, leading to different names for priors with identical properties.^2^ In this study, prior distribution is first named in terms of accuracy—Aligned prior, Divergent prior, and Diffuse prior—to represent accurate, inaccurate, and neutral priors, respectively.^3^ Descriptive phrases were then added to Aligned and Divergent priors to indicate level of informativeness. Five types of priors were defined: (i) Aligned prior with small variance (AlignedSV)—an accurate and informative prior, indicating correct prior belief with certainty; (ii) Aligned prior with large variance (AlignedLV)—an accurate and weakly informative prior, indicating correct prior belief but with low certainty; (iii) Divergent prior with small variance (DivergentSV)—an informative but inaccurate prior, indicating incorrect prior knowledge but with certainty; (iv) Divergent prior with large variance (DivergentLV)—an inaccurate and weakly informative prior, indicating incorrect prior knowledge with low certainty; and (v) Diffuse prior—a neutral and uninformative prior, usually defined with a large variance and prior mean set to zero, indicating no specific prior knowledge about the target parameter.

## Bayesian fit indices

### PP*p*

The posterior predictive *p*-value (PP*p*) is computed as a posterior predictive model-checking (PPMC) procedure for assessing model fit to data. In general, PPMC involves comparing properties of model-generated data with their observed data equivalent. The model is considered to fit the observed data well if the properties of the model-generated data closely match those of the observed data. The extent of mismatch can be represented graphically by comparing plots of the posterior predictive distribution with that of the observed data. Such plots could be a very useful complement to numerical evidence of model fit.^4^ However, it is increasingly common to make determinations about lack of fit or otherwise using what could be likened to a test of statistical significance (Gelman et al., 2013). The degree of mismatch is captured by a discrepancy statistic which forms the basis for developing related specific PPMC procedures.^5^ Such discrepancy statistics could be any statistic^6^ chosen by the researcher (Gelman et al., 1996, 2013; Meng, 1994; Rubin, 1984). The chi-square discrepancy function has become a common summary statistic for measuring global model fit.^7^ Thus, the PP*p* defined below is based on chi-square as a discrepancy statistic.

**Fig. 1:**
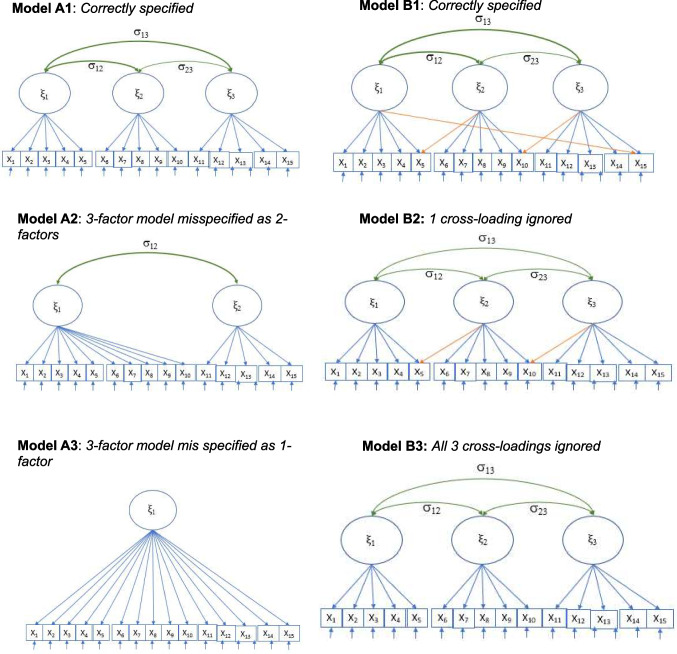
Analysis models

Given a discrepancy function $f({Y}^{obs}, \widehat{\theta })$, where ${Y}^{obs}$ represents observed data, and $\widehat{\theta }$ is a vector of estimated model parameters from the observed data, PP*p* is defined as

4 $$\text{PP}p=P\left(f\left({Y}^{obs}, \widehat{\theta }\right)< f\left({Y}^{rep}, {\widehat{\theta }}_{i}\right) \right)\propto \frac{1}{m}{\sum }_{i=1}^{m}{\gamma }_{i}$$

where $f\left({Y}^{rep},{\widehat{\theta }}_{i}\right)$ represents a discrepancy function realized from the posterior distribution of the *i*^th^ iteration of an MCMC process with *m* total iterations. Here, ${Y}^{rep}$ represents the model-generated or replicated data obtained using the estimated parameter values ${\widehat{\theta }}_{{\varvec{i}}}$. $\text{PP}p$ is then computed as the simple mean of the returned Boolean values (${\gamma }_{i}$) denoted as 1 if $f\left({Y}^{obs}, \widehat{\theta }\right)< f\left({Y}^{rep}, {\widehat{\theta }}_{i}\right)$ and 0 otherwise across *m* iterations (Asparouhov & Muthén, 2010; Gelman et al., 1996; Meng, 1994). A PP*p-*value around 0.5 indicates a good model–data fit, while a PP*p* value less than 0.05 or greater than 0.95 indicates a poor model fit (Asparouhov & Muthén, 2010; Gelman et al., 2013).

PP*p* was found to be less sensitive than likelihood-ratio chi-square test and appropriate for assessing overall model fit with small samples but unreliable for assessing model fit for large samples (*n* $\ge 1000;$ Asparouhov & Muthén, 2010; Hoofs et al., 2018; Lee & Song, 2004; Muthén & Asparouhov, 2012; Rupp et al., 2004).

### BRMSEA, BCFI, and BTLI

Besides PP*p*, two formulations of BRMSEA were recently proposed, namely BRMSEA^ppmc^ (Hoofs et al., 2018) and BRMSEA^DevM^ (Garnier-Villarreal & Jorgensen, 2020).^8^ Both versions of BRMSEA were developed to be analogous to the maximum likelihood-based RMSEA within the frequentist framework (Eq. 5)^9^

5 $$\text{RMSEA}=\sqrt{\text{max}\left(0, \frac{{\chi }^{2}- df}{df(N-1}\right)}$$

where $df={p}^{*}-q$, with ${p}^{*}$= number of unique elements of the sample variance–covariance matrix [*p* (*p* + 1)/2], and $q$ = number of freely estimated parameters. In defining the degree of freedom ($df$), both BRMSEA^ppmc^ and BRMSEA^DevM^ replaced $q$ with the Bayesian equivalent $pD$ (the effective number of parameters; Spiegelhalter et al., 2002). $pD$ is computed by comparing the mean of the posterior deviance ($\overline{D }$) at each *i*th MCMC iteration with the deviance of posterior mean [$D(\overline{\theta })$] as shown in (6).

6 $$pD=\overline{D }- D(\overline{\theta })$$

Consequently, model complexity represented by $df$ was redefined as $df$ = ${p}^{*}-pD$. Note that *pD* $\cong$ $q$ when a noninformative prior is specified. The two versions of BRMSEA differ mainly in the way the elements of the non-centrality parameter ($\lambda ={\chi }^{2}-df$) is defined. The discrepancy function denoting model misfit at iteration *i* of the MCMC process was represented as ${D}_{i}^{obs}-{D}_{i}^{rep}$ in ${\text{BRMSEA}}_{i}^{\text{ppmc}}$ and ${D}_{i}^{obs}- pD$ in ${\text{BRMSEA}}_{i}^{\text{DevM}}$, leading to Eqs. 7 and 8. One commonly used discrepancy function *D* is the chi-square discrepancy function.

7 $${\text{BRMSEA}}_{i}^{\text{ppmc}}=\sqrt{max\left(0, \frac{({D}_{i}^{obs}-{D}_{i}^{rep})-\left(p^*-{p}_{D}\right)}{\left(p^*-{p}_{D}\right)\times N}\right)}$$

8 $${\text{BRMSEA}}_{i}^{\text{DevM}}=\sqrt{\text{max}\left(0, \frac{\left({D}_{i}^{obs}- pD\right)-\left(p^*-{p}_{D}\right) }{{(p}^{*}-pD)\times N}\right)}=\sqrt{\text{max}\left(0, \frac{{D}_{i}^{obs}- {p}^{*}}{{(p}^{*}-pD)\times N}\right)}$$

The formulations of BCFI and BTLI as proposed by Garnier-Villarreal and Jorgensen (2020)^10^ are as shown in Eqs. 9 and 10.

9 $$BCF{I}_{i}=1-\frac{{D}_{H. i}^{obs}- {p}^{*}}{{D}_{o, i}^{obs}- {p}^{*}}$$

10 $$BTL{I}_{i}= \frac{\left({D}_{o,i}^{obs}- {pD}_{o}\right)/\left({p}^{*}- {pD}_{o}\right) -\left({D}_{H, i}^{obs}- {pD}_{H}\right)/\left({p}^{*}- {pD}_{H}\right)}{\left({D}_{o,i}^{obs}- {pD}_{o}\right)/\left({p}^{*}- {pD}_{o}\right)-1}$$

where the subscript “*H*” denotes the hypothesized model, and the subscript “*o*” denotes the null or independent model in which covariances are constrained to zero with mean and variances freely estimated.^11^

Previous studies indicated that when noninformative priors are specified, the recently introduced BSEM fit indices yield similar results as their MLE counterparts across levels of sample sizes, model types, and misspecifications (Garnier-Villarreal & Jorgensen, 2020; Hoofs et al., 2018). However, there is limited research examining the performance of these fit indices when informative priors are specified (Edwards & Konold, 2022; Winter & Depaoli, 2022b).

### Impact of priors on fit indices

Prior choice has both direct and indirect influence on the computation of the newly introduced BSEM fit indices due to the way they were formulated (see Eqs. 7 to 10). Directly, the prior enters the computation of BRMSEA, BCFI, and BTLI through the effective number of parameters (*pD*). When a noninformative prior is specified, *pD* is approximately equal to *q*. However, *pD* is increasingly less than *q* as the prior informativeness increases^12^ (Garnier-Villarreal & Jorgensen, 2020). Indirectly, the effect of prior choice on BSEM fit indices is mediated through the realized posterior distribution which is a function of the prior and data likelihood. It is reasonable to expect that these direct and indirect effects of priors on BSEM fit indices could in turn affect the fit indices’ ability to serve as an evaluation tool. Such effects are yet to be sufficiently investigated.

## Literature review and the study purpose

Hoofs et al. (2018) introduced and assessed the performance of the BRMSEA^ppmc^ in BCFA, using both informative and noninformative priors. They found that in terms of rejection rate and precision, measured by the 90% credible interval,^13^ accurate informative priors performed marginally better than diffuse priors, and that an inaccurate prior (the same as DivergentSV)^14^ resulted in the poorest performance. Garnier-Villarreal and Jorgensen (2020) assessed the adequacy of BRMSEA^DevM^, along with BCFI, BTLI, BNFI, B $\widehat{\Gamma } ,$ and BM_C_, using noninformative priors, and called for systematic evaluation of these fit indices using informative priors. Winter and Depaoli (2022b) investigated prior sensitivity of BRMSEA^DevM^, along with BCFI and BTLI, using both informative and noninformative priors in the context of latent growth models with sample sizes not exceeding 500. Their result indicated that in terms of average model fit and rejection rate, BCFI and BTLI comparably indicated poorer fit with divergent priors relative to diffuse or aligned priors. BRMSEA performed comparatively worse than BCFI and TLI across all prior types when *n* < 500. Liang (2020) investigated prior sensitivity of BRMSEA, BCFI, and BTLI along with deviance information criterion (DIC), Bayesian information criterion (BIC), and PP*p*, focusing on the impact of small-variance priors assigned to cross-loadings in correctly specified models. Results indicated that BCFI, BTLI, and BRMSEA were insensitive to choices of both noninformative/vague priors and shrinkage priors, but that their performance improved across board as primary loadings and prior variances increased. Edwards and Konold (2022) investigated the impact of varying informative prior specifications with model misspecification on BRMSEA, BCFI, and BTLI within Bayesian CFA. However, their simulation models were limited in complexity, variation in model size, and the types of misspecifications examined (i.e., two latent factors, one cross-loading, all primary loadings fixed to 0.7). Cao et al. (2023) examined the sensitivity of Bayesian fit indices with results applicable only to structural misspecification. They found that BRMSEA, BTLI, and BCFI were not sensitive to structural misspecification.

The behavior of these BSEM fit indices with more complex models, and varying item loading magnitudes, as common in practice, has not yet received sufficient attention. Literature indicates that in the frequentist framework, the behavior of fit indices is influenced by model size. For example, when there are more observed items in the SEM model, CFI and RMSEA are less sensitive to model misspecification (Kenny & McCoach, 2003). These global fit indices flag higher-quality measurement models as having poorer fit than lower-quality measurement models with the same level of structural misspecification. This was termed the reliability paradox and was investigated within the Bayesian framework by Konold and Sanders (2023). They found that irrespective of prior type, BRMSEA indicated worsening fit as the magnitude of factor loading increased, thus exhibiting the reliability paradox. They also found that although BCFI and BTLI appeared to perform well with complex correctly specified models, they incorrectly indicated good fit for severely misspecified models with high factor loadings (about 0.80). However, the complexity of the models employed by Konold and Sanders has, at the maximum, three latent factors with five items per factor and did not consider cross-loadings. The field will benefit from knowing more about the behavior of the newly introduced BSEM fit indices in more complex models with more variation in prior specification as may be encountered in practice. Our study systematically evaluated the prior sensitivity of the newly introduced BSEM fit indices, including BRMSEA, BCFI, and BTLI, and PP*p*, using a wide range of sample sizes, priors of varying informativeness and accuracy, latent factor and cross-loading of varying magnitudes, and models with varying sizes and levels of misspecification.

To avoid the effect of model complexity on the performance of the BSEM fit measures being confounded with the effect of other combinations of conditions under investigation, the present study was conducted in two parts. Study 1 investigated prior sensitivity of Bayesian SEM fit indices to latent factor and cross-loading misspecifications for three-factor models with various prior types, sample sizes, latent factor correlations, and cross-loading magnitudes. Based on the findings from study 1, study 2 employed selected conditions in study 1, to investigate the impact of model complexity on the sensitivity of Bayesian SEM fit indices. By this design, study 2 enabled us to explore how varying model complexity affects the prior sensitivity of BSEM fit indices to model misspecifications. Table A8 in the appendix provides an overview of literature on the evaluation of BRMSEA, BCFI, and BTLI with respect to major findings and associated model and prior types.

## Study 1: Prior sensitivity of BSEM fit measures to model misspecification 

### Population data generation

Study 1 investigated the prior sensitivity of BRMSEA, BCFI, BTLI, and PP*p* to different degrees of dimensionality and cross-loading misspecifications. Two main models were used for population data generation, designated as models A and B. Model A is an independent cluster CFA model with three latent factors (${\xi }_{1}$, ${\xi }_{2}$, ${\xi }_{3}$), each measured with five continuous indicators with relatively strong primary factor loadings (between 0.7 and 0.8) to mimic empirical research (Garrido et al., 2016; Hair et al., 2017; Shi et al., 2019). The latent factor correlations for model A were manipulated at 0.85 and 0.35, representing strong and weak factor correlations. Model B differs from model A by having fixed factor correlation (0.5) and three items with cross-loadings. The cross-loadings (${\uplambda }_{\text{c}}$) were manipulated at 0.2 and 0.5 to represent small and large cross-loadings (Kline, 2015). All latent factors were standardized for both models.

11 $${\Lambda }_{\text{A}}{\prime}=\left(\begin{array}{c}.70\ .70\ .75\ .80\ .80\ 0\ 0\ 0\ 0\ 0\ 0\ 0\ 0\ 0\ 0\\ 0\ 0\ 0\ 0\ 0\ .70\ .70\ .75\ .80\ .80\ 0\ 0\ 0\ 0\ 0\\ 0\ 0\ 0\ 0\ 0\ 0\ 0\ 0\ 0\ 0\ .70\ .70\ .75\ .80\ .80\end{array}\right)$$

12 $${\Lambda }_{\text{B}}{\prime}=\left(\begin{array}{c}.70\ .70\ .75\ .80\ .80\ 0\ 0\ 0\ 0\ 0\ 0\ 0\ 0\ 0\ {\uplambda }_{\text{c}}\\ 0\ 0\ 0\ 0\ {\uplambda }_{\text{c}} .70\ .70\ .75\ .80\ .80\ 0\ 0\ 0\ 0\ 0 \\ 0\ 0\ 0\ 0\ 0\ 0\ 0\ 0\ {\uplambda }_{\text{c}} 0\ .70\ .70\ .75\ .80\ .80\end{array}\right)$$

Four levels of sample sizes were considered, 75, 200, 500, and 2000, representing small (75, 200), moderate (500), and large (2000) sample sizes. The chosen sample sizes are commonly encountered in empirical research. Small sample sizes are common in education and psychology research, as collecting sufficient data can be challenging on some occasions (Pedersen & Watt, 2014). Overall, study 1 encompassed 16 data generation conditions: model A (2 latent factor correlation magnitudes × 4 sample sizes) and model B (2 item cross-loading magnitudes × 4 sample sizes).

### Analysis

Three models were analyzed for model A to investigate prior sensitivity of BRMSEA, BCFI, BTLI, and PP*p* to dimensionality misspecification. The first model was the correctly specified model with three factors, and the second and third were misspecified as two-factor and one-factor models, respectively (see Fig. 1, models A1 to A3).

Similarly, three analysis models were examined for model B to investigate prior sensitivity of the fit indices to cross-loading misspecification. The first model was correctly specified with three cross-loadings, and the second and third were misspecified by ignoring one and all three cross-loadings, respectively (Fig. 1, models B1 to B3). For all analysis models, latent factor variances were constrained to 1, while factor correlations and item loadings were freely estimated. Analyses were conducted at both the population and sample levels. At the population level, population fit indices for both correctly specified and misspecified models were computed by fitting a large data set, *N* = 1,000,000 (Shi & Maydeu-Olivares, 2020), to the population covariance matrix. At the sample level, a simulation study was conducted to examine prior sensitivity of the fit indices to dimensionality and cross-loading misspecification across chosen sample sizes.

### Prior specification

Five normal priors *N*(*µ*, *σ*^*2*^) were implemented on the factor loadings:

(1) Diffuse prior: *λ* ~ *N*(0, 1000), (2) Aligned prior with small variance (AlignedSV): *λ* ~ *N*(${\mu }_{\lambda }$, 0.01), where mean (${\mu }_{\lambda }$) was the population factor loading values, (3) Aligned prior with large variance (AlignedLV): *λ* ~ *N*(${\mu }_{\lambda }$, 1), (4) Divergent prior with small variance (DivergentSV): *λ* ~ *N*(0.5, 0.01), where the mean was set to be two to three standard deviations away from the mean of population factor loadings, and (5) Divergent prior with large variance (DivergentLV): *λ* ~ *N*(0.5, 1). This range of priors was chosen to provide opportunity for a systematic investigation of BSEM fit indices responses to various levels of prior informativeness and accuracy. Besides the prior on factor loadings, M*plus* noninformative default priors were assigned to intercepts τ~ *N*(0, $\infty$), residual variances δ~ *IG*(− 1, 0), and latent factor covariances Φ ~ *IW*(0, − 7)^15^ (M*plus* 8.7; Muthén and Muthén, 1998–2021). Though inverse gamma prior and inverse Wishart prior are commonly specified as noninformative prior for variance and covariance parameters, respectively, it is worth noting that these priors have been shown to become unintentionally informative in instances where the variance/covariance parameter is close to zero (Gelman, 2006; Schuurman et al., 2016). Therefore, these priors should be used cautiously when variance or covariances are the parameters of interest.

### BSEM implementation

Each of the 16 data conditions was analyzed with three models, and each analysis model had five prior specifications, totaling 240 unique combinations of conditions. Normally distributed data with 500 replications for each of the 240 analysis conditions were generated and analyzed using SAS 9.4 (SAS Institute Inc., 2002-2020) and M*plus* 8.7 (Muthén & Muthén, 1998-2021). A Bayes estimator with a Gibbs sampler was used for drawing posterior samples from three MCMC chains, with a minimum of 10,000 and a maximum of 500,000 iterations. An M*plus* BCONVERGENCE default value of 0.05 was used; hence, convergence was determined by the potential scale reduction factor^16^ (PSR) < 1.10 (Asparouhov & Muthén, 2010; Gelman & Rubin, 1992). The use of three MCMC chains and relatively high minimum/maximum iteration set to 10,000/500,000 was aimed at increasing the convergence rate and the reliability of realized posterior distribution of each parameter. The first half of iterations were discarded as burn-ins, and the second half was used for summarizing Bayesian statistics. With a minimum of 10,000 samples across three chains, at least 15,000 samples (10,000/2 × 3) were used for Bayesian inference. The first 500 replications were used for computing the convergence rates. If non-convergence occurred during the first 500 analyses, more replications of data were generated and analyzed until 500 converged solutions were obtained for each unique condition.^17^

### Outcome evaluation

At the sample level, the strength of the association between BSEM model fit indices (BRMSEA, BCFI, BTLI, and PP*p*) and the study design facets were evaluated using factorial analysis of variance (ANOVA). The posterior mean and standard deviation of all fit measures were examined. All fit indices were also evaluated using rejection rates (false and true positive rates). False positive rate was defined as the proportion of replications in which the fit indices signaled poor model fit, based on the cutoff criteria below, when the model was in fact correctly specified. True positive rate was defined as the proportion of replications in which the fit indices correctly flagged poor model fit when the model was misspecified. PP*p* values less than 0.05 and PP*p* values greater than 0.95, BRMSEA greater than 0.06, and BCFI/BTLI values less than 0.95 were indications of inadequate model fit (Hu & Bentler, 1999; Muthén & Asparouhov, 2012).

### Results for study 1

#### Convergence rate

The convergence rate was 100% or approximately 100% for most models across different sample sizes and prior types. However, the three-factor model with correlation 0.35, misspecified as a two-factor model (Fig. 1, model A2), yielded relatively lower convergence rate (ranging from 66% to 72%) compared to all other models when sample size was large (*n* = 2,000) (see Table A1 in the Appendix). It appeared that as the sample size increased, the unequal number of items per factor and increasing severity of misspecification lowered the convergence rates.

**Table 1:** Eta-squared values for ANOVA results [median effect size (${\upeta }^{2}$) $=$ 0.0588]

Population model	Effect	BRMSEA	BCFI	BTLI	PP*p*		BRMSEA	BCFI	BTLI	PP*p*
		Study 1					Study 2			
Model A						Model C				
	# Factors						.016	.023	.017	.000
	# Items						.001	.049	.045	.001
	*n*	.012	.003	.002	.003		.041	.041	.040	.000
	Prior	.007	.002	.001	.034		**.173**	**.174**	**.173**	**.165**
	CorMg	**.242**	**.365**	**.368**	.000					
	Lmis	**.513**	**.357**	**.357**	**.574**		**.480**	**.471**	**.481**	**.351**
	CorMg × Lmis	**.156**	**.240**	**.239**	.010					
	Prior × Lmis						**.128**	**.100**	**.102**	**.165**
Model B						Model D				
	# Cld						.013	.022	.018	.000
	# Items						.010	.013	.014	.002
	CldMg	**.184**	**.106**	**.119**	.030					
	Cldmis	**.327**	**.193**	**.211**	**.426**		**.317**	**.222**	**.228**	**.368**
	Prior	**.072**	**.127**	**.119**	**.052**		**.360**	**.453**	**.450**	**.154**
	*n*	**.105**	**.131**	**.120**	.016		**.082**	**.081**	**.080**	.001
	Prior × cldmiss						.032	.018	.019	**.151**
	*n* × Prior	**.086**	**.216**	**.194**	.020					
	cldMg × cldmis	**.108**	**.102**	**.113**	.020					

CorMg: correlation magnitude; Lmis: latent factor misspecification; cldMg: cross-loading magnitude; cldmis: cross-loading misspecification

***Study 1 models:*** Model A: 3 factors, 5 items each; correlation (0.35 vs. 0.85); Model B: 3 factors, 5 items each; cross-loading magnitude (0.2 vs. 0.5)

***Study 2 models:*** Model C: 3 or 6 factors; 5 or 10 items each; Model D: 3 or 6 factors; 5 or 10 items each; and 6 or 12 items cross-loading for each factor

#### Analysis of variance

Factorial ANOVA was used to evaluate the contribution of each study design facet in explaining the sampling variance observed in the fit indices. Design factors that have an eta-squared value $\left({\eta }^{2}\right)$ greater than 0.0588 (moderate effect size) or 0.1379 (large effect size) (Cohen, 1973), were highlighted as having an important impact on the associated fit index. The result, as shown in Table 1, indicated that among the highlighted values for model A, about 16% to 57% of the variation in the fit indices can be attributed to latent factor misspecification (${\eta }^{2}$ ranging from 0.156 for BRMSEA to 0.574 for PP*p*). Also, for model A, the interaction between the magnitude of the factor correlation and latent factor misspecification indicated large effects for BRMSEA, BCFI, and BTLI (${\eta }^{2}$ = 0.156 to 0.368) but showed little effect on PP*p*. For model B, cross-loading magnitude, cross-loading misspecification (Cldmis), and their interaction had a large effect on BRMSEA, BCFI, and BTLI. For PP*p*, only Cldmis indicated a large effect.

Overall, for study 1, prior type, sample sizes, and their interaction seemed to have a negligeable effect on fit values of models with misspecified latent factors (model A), but in contrast, these design factors indicated large effect for most of the fit measures on models with cross-loading misspecifications (model B).

#### Population fit value

Table 2 (models A and B) presents population-level model fit values for BRMSEA, BCFI, and BTLI. As expected, the result indicated that model fit worsened as misspecification became more severe. The result also indicated that at a given level of misspecification, all fit indices increasingly signal better fit as cross-loading magnitude decreased and as latent factor correlation magnitude increased, holding other design facets constant.

**Table 2:** Population RMSEA, CFI, and TLI values for study 1 and study 2

	**Factor correlation = 0.85**			**Factor correlation = 0.35**		
Model	RMSEA	CFI	TLI	RMSEA	CFI	TLI
A1 (A33_cor.35/.85)	0.000	1.000	1.000	0.000	1.000	1.000
A2 (A32_cor.35/.85)	0.054	0.969	0.964	0.143	0.732	0.684
A3 (A31_cor.35/.85)	0.073	0.942	0.932	0.193	0.502	0.419
**C1-1 (F*3-I*10*, crt3*)**	0.000	1.000	1.000	0.000	1.000	1.000
C1-2 (F3-*I*10*, mis2*)	0.054	0.940	0.936	0.112	0.710	0.688
**C1-3 (F3*-I*10*, mis1*)**	0.062	0.920	0.914	0.149	0.487	0.448
**C2-1 (F6*-I*5*, crt6*)**	0.000	1.000	1.000	0.000	1.000	1.000
C2-2 (F6-*I*5*, mis4*)	0.047	0.952	0.948	0.115	0.619	0.585
**C2-3 (F6-*I*5*, mis2*)**	0.049	0.947	0.943	0.129	0.516	0.479
**C3-1 (F6-*I*10*, crt6*)**	0.000	1.000	1.000	0.000	1.000	1.000
C3-2 (F6-*I*10*, mis4*)	0.037	0.943	0.941	0.078	0.711	0.700
**C3-3 (F6-*I*10*, mis2*)**	0.043	0.923	0.921	0.103	0.493	0.475
	**Item cross-loading = 0.5**			**Item cross-loading = 0.2**		
B1 (B33_cld.2 /.5)	0.000	1.000	1.000	0.000	1.000	1.000
B2 (B32_cld.2 /.5)	0.073	0.955	0.944	0.024	0.994	0.992
B3 (B30_cld.2 /.5)	0.117	0.881	0.857	0.041	0.981	0.977
**D1-1 (F3-*I*10-cld6, 6)**	0.000	1.000	1.000	0.000	1.000	1.000
D1-2 (F3-*I*10-cld6, 4)	0.032	0.986	0.984	0.014	0.997	0.997
**D1-3 (F3-*I*10-cld6, 0)**	0.041	0.976	0.974	0.020	0.994	0.993
**D2-1 (F6-*I*5-cld6, 6)**	0.000	1.000	1.000	0.000	1.000	1.000
D2-2 (F6-*I*5-cld6, 4)	0.021	0.994	0.993	0.009	0.999	0.998
**D2-3 (F6-*I*5-cld6, 0)**	0.034	0.982	0.980	0.016	0.996	0.995
**D3-1 (F6-*I*10-cld12, 12)**	0.000	1.000	1.000	0.000	1.000	1.000
D3-2 (F6-*I*10-cld12, 8)	0.021	0.987	0.986	0.010	0.997	0.997
**D3-3 (F6-*I*10-cld12, 0**)	0.028	0.978	0.977	0.014	0.994	0.994

Bold font indicates models used for sample-level analysis in study 2

Models A1–A3 and B1–B3 are from study 1, while C and D models were used in study 2

For study 1: models were named as follows: cor = correlation and cld = cross-loading. Hence, A33_cor.35/cor.85 = three-factor model, correctly specified with factor correlation varied at 0.35 and 0.85. A32_cor.35/cor.85 = three-factor model, misspecified as two factors with factor correlation varied at 0.35 and 0.85; B33_cld.2/cld.5 = three cross-loadings, correctly specified with cross-loading magnitude varied at .2 and .5; B32_cld.2/cld.5 = three cross-loadings, misspecified by ignoring one, with cross-loading magnitude varied at .2 and .5; and so on

For study 2: models were named as follows: F = number of latent factors, I = number of items, crt = correct specification, mis = misspecification, and cld = number of cross-loadings. For example, “F3-I5, crt3” reads as three-factor model with five items correctly specified as a three-factor model, and “F3-I5, mis2” reads as three-factor model with five items misspecified as a two-factor model. Similarly, “F6-I10-cld12, 8” should be read as a six-factor model with ten items and 12 cross-loadings misspecified as having eight cross-loadings

#### Sample statistics of fit measures

Sample-level results (Tables 3 and 4) indicated that the average fit indices values for BRMSEA, BCFI, and BTLI were negatively biased, relative to their population values, when the sample size was small (*n* < 500). However, the extent of bias decreased as sample size increased. Little or no bias was evident with sample size of 2,000. BRMSEA, BCFI, and BTLI showed the greatest bias when an informative but inaccurate prior (DivergentSV) was specified. The remaining four prior types produced less negatively biased fit values compared to DivergentSV. Tables A6 and A7 in the Appendix present summaries of sample statistics for PP*p*, with no population values available for comparison. It revealed that in a few instances, PP*p* signaled good model fit for misspecified models with *n* < 500. This happened more often when cross-loading magnitude was low (0.2) and when factor correlation was high (0.85), leading to relatively less severe model misspecification.

**Table 3:** Study 1: Mean (*SD*) of BRMSEA, BCFI, and BTLI by sample size, prior specification, covariance structure and model factor misspecification

	***n***	**Prior type**	**Three factors misspecified as one (Model A3)**		**Three factor misspecified as two (Model A2)**		**Three factors correctly specified (Model A1)**	
			**A31_cor.35**	**A31_cor.85**	**A32_cor.35**	**A32_cor.85**	**A33_cor.35**	**A33_cor.85**
**Population BRMSEA**			**0.193**	**0.073**	**0.143**	**0.054**	**0.000**	**0.000**
*BRMSEA*	75	DivergentSV	0.19 (0.02)	0.09 (0.02)	0.15 (0.02)	0.08 (0.02)	**0.10** (0.04)	**0.08** (0.02)
		Other four priors	0.19 (0.02)	0.08 (0.02)	0.14 (0.02)	0.07 (0.02)	0.04 (0.03)	0.04 (0.02)
	200	DivergentSV	0.19 (0.01)	0.08 (0.01)	0.14 (0.01)	0.07 (0.01)	0.04 (0.01)	**0.07** (0.01)
		Other four priors	0.19 (0.01)	0.07 (0.01)	0.14 (0.01)	0.06 (0.01)	0.02 (0.02)	0.02 (0.02)
	500	DivergentSV	0.19 (0.01)	0.07 (0.01)	0.14 (0.01)	0.07 (0.01)	0.02 (0.01)	**0.07** (0.01)
		Other four priors	0.19 (0.01)	0.07 (0.01)	0.14 (0.01)	0.05 (0.01)	0.01 (0.01)	0.01 (0.01)
	2000	DivergentSV	0.19 (0.00)	0.07 (0.00)	0.14 (0.00)	0.06 (0.00)	0.01 (0.00)	0.01 (0.00)
		Other four priors	0.19 (0.00)	0.07 (0.00)	0.14 (0.00)	0.05 (0.00)	0.00 (0.00)	0.00 (0.00)
**Population BCFI**			**0.502**	**0.942**	**0.732**	**0.969**	**1.000**	**1.000**
*BCFI*	75	DivergentSV	0.50 (0.07)	0.91 (0.03)	0.69 (0.07)	0.92 (0.03)	**0.85 (0.10)**	**0.92 (0.03)**
		Other four priors	0.51 (0.07)	0.92 (0.03)	0.74 (0.05)	0.95 (0.03)	0.97 (0.02)	0.98 (0.02)
	200	DivergentSV	0.51 (0.05)	0.93 (0.02)	0.73 (0.03)	0.94 (0.02)	0.98 (0.01)	0.94 (0.02)
		Other four priors	0.51 (0.04)	0.94 (0.02)	0.75 (0.03)	0.97 (0.01)	0.99 (0.01)	0.99 (0.01)
	500	DivergentSV	0.51 (0.03)	0.94 (0.01)	0.74 (0.02)	0.94 (0.01)	1.00 (0.00)	0.95 (0.01)
		Other four priors	0.51 (0.03)	0.94 (0.01)	0.74 (0.02)	0.97 (0.01)	1.00 (0.00)	1.00 (0.00)
	2000	DivergentSV	0.50 (0.01)	0.94 (0.00)	0.74 (0.01)	0.97 (0.00)	1.00 (0.00)	1.00 (0.00)
		Other four priors	0.50 (0.01)	0.94 (0.00)	0.74 (0.01)	0.97 (0.00)	1.00 (0.00)	1.00 (0.00)
**Population BTLI**			**0.419**	**0.932**	**0.684**	**0.964**	**1.000**	**1.000**
*BTLI*	75	DivergentSV	0.47 (0.07)	0.91 (0.03)	0.66 (0.08)	0.91 (0.03)	**0.83 (0.12)**	**0.92 (0.03)**
		Other four priors	0.44 (0.07)	0.91 (0.04)	0.70 (0.06)	0.94 (0.03)	0.97 (0.03)	0.97 (0.02)
	200	DivergentSV	0.46 (0.05)	0.93 (0.02)	0.70 (0.03)	0.93 (0.02)	0.98 (0.01)	**0.93 (0.02)**
		Other four priors	0.43 (0.05)	0.93 (0.02)	0.70 (0.03)	0.96 (0.02)	0.99 (0.01)	0.99 (0.01)
	500	DivergentSV	0.44 (0.03)	0.93 (0.01)	0.70 (0.02)	0.94 (0.01)	0.99 (0.00)	0.94 (0.01)
		Other four priors	0.43 (0.03)	0.93 (0.01)	0.70 (0.02)	0.96 (0.01)	1.00 (0.00)	1.00 (0.00)
	2000	DivergentSV	0.43 (0.02)	0.93 (0.01)	0.69 (0.01)	0.96 (0.00)	1.00 (0.00)	1.00 (0.00)
		Other four priors	0.42 (0.02)	0.93 (0.01)	0.69 (0.01)	0.96 (0.00)	1.00 (0.00)	1.00 (0.00)

A33_cor.85 = three-factor model, not misspecified; A32_cor.85 = three-factor model, misspecified as two factors. A31_cor.85 = three-factor model, misspecified as one factor; A33_cor.35 = three-factor model, not misspecified A32_cor.35 = three-factor model, misspecified as two factors; A31_cor.35 = three-factor model, misspecified as one factor

DivergentSV = Divergent with small variance; other four priors: AlignedLV, AlignedSV, DivergentLV, and Diffuse prior

AlignedLV = Aligned with large variance; AlignedSV = Aligned with small variance; DivergentLV = Divergent with large variance

**Table 4:** Study 1: Mean (*SD*) of BRMSEA, BCFI, and BTLI by sample size, prior specification, cross-loading magnitude, and cross-loading misspecification

			**All three cross-loadings ignored (Model B3)**		**One cross-loading** **ignored (Model B2)**		**Three cross-loadings correctly specified (Model B1)**	
	***n***	**Prior type**	**B30_cld.2**	**B30_cld.5**	**B32_cld.2**	**B32_cld.5**	**B33_cld.2**	**B33_cld.5**
**Population BRMSEA**			**0.041**	**0.117**	**0.024**	**0.073**	**0.000**	**0.000**
*BRMSEA*	75	DivergentSV	0.15 (0.02)	0.18 (0.02)	0.14 (0.02)	0.17 (0.02)	**0.14 (0.03)**	**0.13 (0.04)**
		Other four priors	0.05 (0.02)	0.12 (0.02)	0.05 (0.03)	0.08 (0.02)	0.04 (0.03)	0.04 (0.03)
	200	DivergentSV	0.07 (0.03)	0.17 (0.02)	0.05 (0.02)	0.09 (0.02)	0.04 (0.02)	0.04 (0.01)
		Other four priors	0.04 (0.01)	0.12 (0.01)	0.03 (0.02)	0.07 (0.01)	0.01 (0.01)	0.02 (0.01)
	500	DivergentSV	0.05 (0.01)	0.12 (0.01)	0.03 (0.01)	0.08 (0.01)	0.02 (0.01)	0.02 (0.01)
		Other four priors	0.04 (0.01)	0.12 (0.01)	0.02 (0.01)	0.07 (0.01)	0.01 (0.01)	0.01 (0.01)
	2000	DivergentSV	0.04 (0.00)	0.12 (0.00)	0.02 (0.00)	0.07 (0.00)	0.01 (0.00)	0.01 (0.00)
		Other four priors	0.04 (0.00)	0.12 (0.00)	0.02 (0.00)	0.07 (0.00)	0.00 (0.00)	0.00 (0.00)
**Population BCFI**			**0.981**	**0.881**	**0.994**	**0.955**	**1.000**	**1.000**
*BCFI*	75	DivergentSV	0.72 (0.06)	0.68 (0.05)	0.75 (0.07)	0.73 (0.06)	**0.77 (0.07)**	**0.83 (0.09)**
		Other four priors	0.96 (0.03)	0.87 (0.03)	0.97 (0.02)	0.94 (0.03)	0.98 (0.02)	0.98 (0.02)
	200	DivergentSV	0.94 (0.05)	0.74 (0.05)	0.96 (0.04)	0.93 (0.03)	0.98 (0.02)	0.99 (0.01)
		Other four priors	0.98 (0.01)	0.88 (0.02)	0.99 (0.01)	0.95 (0.01)	1.00 (0.01)	1.00 (0.01)
	500	DivergentSV	0.98 (0.01)	0.88 (0.01)	0.99 (0.00)	0.95 (0.01)	1.00 (0.01)	1.00 (0.01)
		Other four priors	0.98 (0.01)	0.88 (0.01)	0.99 (0.00)	0.95 (0.01)	1.00 (0.01)	1.00 (0.01)
	2000	DivergentSV	0.98 (0.00)	0.88 (0.01)	0.99 (0.00)	0.95 (0.00)	1.00 (0.01)	1.00 (0.01)
		Other four priors	0.98 (0.00)	0.88 (0.01)	0.99 (0.00)	0.96 (0.00)	1.00 (0.01)	1.00 (0.01)
**Population BTLI**			**0.977**	**0.857**	**0.992**	**0.944**	**1.000**	**1.000**
*BTLI*	75	DivergentSV	0.70 (0.07)	0.65 (0.05)	0.73 (0.07)	0.70 (0.07)	**0.75 (0.08)**	**0.81 (0.09)**
		Other four priors	0.95 (0.03)	0.84 (0.04)	0.96 (0.03)	0.92 (0.03)	0.97 (0.03)	0.98 (0.02)
	200	DivergentSV	0.92 (0.06)	0.70 (0.05)	0.96 (0.05)	0.91 (0.04)	0.97 (0.03)	0.98 (0.01)
		Other four priors	0.97 (0.01)	0.85 (0.02)	0.99 (0.01)	0.94 (0.01)	0.99 (0.01)	1.00 (0.01)
	500	DivergentSV	0.97 (0.01)	0.85 (0.01)	0.99 (0.01)	0.94 (0.01)	0.99 (0.00)	1.00 (0.00)
		Other four priors	0.98 (0.01)	0.85 (0.01)	0.99 (0.00)	0.94 (0.01)	1.00 (0.00)	1.00 (0.00)
	2000	DivergentSV	0.98 (0.00)	0.85 (0.01)	0.99 (0.00)	0.94 (0.00)	1.00 (0.00)	1.00 (0.00)
		Other four priors	0.98 (0.00)	0.86 (0.01)	0.94 (0.00)	1.00 (0.00)	1.00 (0.00)	

B33_cld.2 = three cross-loadings, non-misspecified; B32_cld.2 = three cross-loadings, one misspecified

B30_cld.2 = three cross-loading, all three misspecified; B33_cld.5 = three cross-loadings, non-misspecified

B32_cld.5 = three cross-loadings, one misspecified; B30_cld.5 = three cross-loading, all three misspecified

DivergentSV = Divergent with small variance; other four priors: AlignedLV, AlignedSV, DivergentLV, and Diffuse prior

AlignedLV = Aligned with large variance; AlignedSV = Aligned with small variance; DivergentLV = Divergent with large variance

#### False and true positive rates

**Fig. 2:**
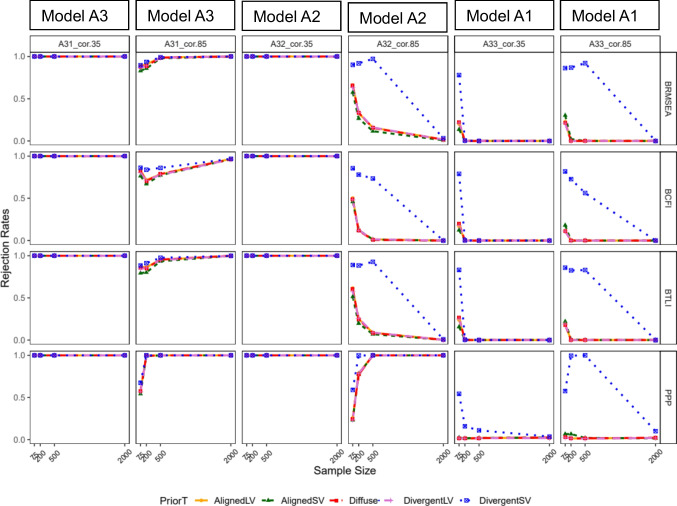
Study 1: Rejection rate across prior specification, sample size, and model latent factor misspecification for BRMSEA, BCFI, BTLI, and PPp. *Note:* Criteria: Model with poor fit defined as BRMSEA > 0.06, BCFI < 0.95 A33, BTLI < 0.95, PPP < 0.05, & PPPP < 0.05. A33_cor.85 = three-factor model, not misspecified; A32_cor.85 = three-factor model, misspecified as two factors. A31_cor.85 = three-factor model, misspecified as one factor; A33_cor.35 = three-factor model, not misspecified. A32_cor.35 = three-factor model, misspecified as two factors; A31_cor.35 = three-factor model, misspecified as one factor. AlignedLV = Aligned with large variance; AlignedSV = Aligned with small variance. DivergentLV = Divergent with large variance; DivergentSV = Divergent with small variance

Figure 2 presents rejection rates for each fit index given a specified prior, sample size, and varying levels of latent factor misspecifications. Columns 1 to 4 represent different levels of misspecification, while the last two columns (columns 5 and 6) contain correctly specified models. The results indicated that all fit indices performed well with low latent factor correlation (0.35). True positive rates for these models were approximately 100% for all prior types and sample sizes (columns 1 and 3 of Fig. 2). However, under less severe latent factor misspecification (column 4, factor correlation of 0.85), the true positive rate decreased across all prior types, as the sample size increased, except for PP*p*, which showed an increase in true positive rate with larger sample size for all prior types. Columns 5 and 6 indicated that the false positive rates were nearly zero for all fit indices across prior types when sample size was sufficiently large (*n* $\ge$ 200). In relatively small samples, false positive rates were the highest when DivergentSV was specified, with inflated rates up to about 80%. For other fit indices, false positive rates were inflated to around 20% only when *n* $\le$ 200.

**Fig. 3:**
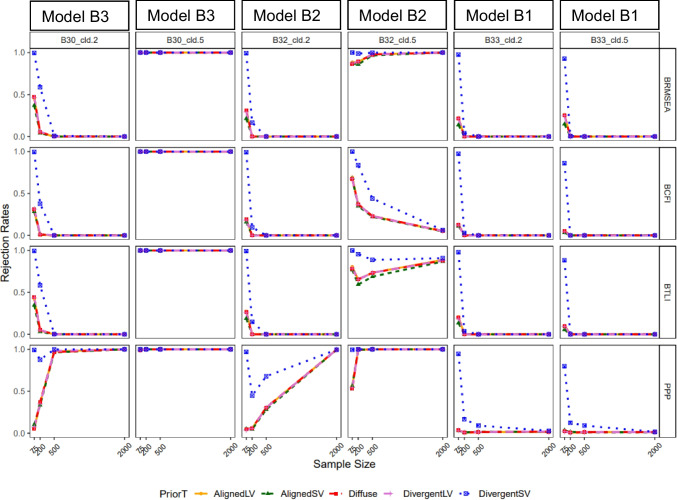
Study 1: Rejection rate across prior specification, sample size, and cross-loading misspecification for BRMSEA, BCFI, and BTLI, and PPp. *Note:* Criteria: Model with poor fit defined as BRMSEA > 0.06, BCFI < 0.95 A33, BTLI < 0.95, PPP < 0.05, & PPPP < 0.05. B33_cld.2 = three cross-loadings, non-misspecified; B32_cld.2 = three cross-loadings, one misspecified. B30_cld.2 = three cross-loading, all three-misspecified; B33_cld.5 = three cross-loadings, non-misspecified. B32_cld.5 = three cross-loadings, one misspecified; B30_cld.5 = three cross-loading, all three-misspecified. AlignedLV = Aligned with large variance; AlignedSV = Aligned with small variance; DivergentLV = Divergent with large variance; DivergentSV = Divergent with small variance

Figure 3 presents the rejection rates for model B with varying levels of cross-loading misspecifications. In general, true positive rates for BRMSEA, BCFI, and BTLI decreased with lower cross-loading magnitude and larger sample size across all prior types. However, for PP*p*, true positive rate increased as sample size increased. For all fit measures, when *n* $\le$ 200, false positive rate increased to about 20% for all priors except for DivergentSV, which showed inflated false positive rates to about 80% or more (see columns 5 and 6 of Fig. 3). Figure 3 shows a dip in two conditions of model B2 for BTLI and PP*p*. This dip might indicate instability or sensitivity of the fit indices to specific combinations of sample sizes, level of misspecification, and prior distributions. For smaller sample sizes, the prior might have an outsized effect, which could lead to unexpected variations such as dips, as the model might struggle to capture the data structure adequately.

### Summary of findings for study 1

Overall, power to detect misspecification increased with the severity of the misspecification. Fit indices performed comparably across prior types, except for DivergentSV, which showed greater model misfit due to misaligned prior information. While DivergentSV demonstrated higher power, it inflated type I error rates with small samples. PP*p* provided the best power for detecting mild misspecifications while maintaining good type I error control for larger samples.

### Study 2: The impact of model size

#### Design factors for data generation

Study 2 was designed to investigate the impact of model size on prior sensitivity of Bayesian SEM fit indices. Models C and D, each containing three sets of data-generating models, were used. The first three models (C1, a three-factor model; C2 and C3, both six-factor models) were used to investigate the impact of model size with latent factor misspecification, while the second set of three models (D1, a three-factor model; D2 and D3, both six-factor models) were used to investigate the impact of model size with cross-loading misspecification (see Table 5 for other design facets). Primary factor loadings varied from 0.7 to 0.8 as in study 1. Latent factor correlations were fixed to 0.85 for all models, while cross-loading magnitudes were fixed to 0.5 for model D. To focus on model complexity, relatively small sample sizes (200 and 500) were used. All other listed design facets were chosen because they elicited higher prior sensitivity for the fit measures in study 1. Resultantly, they provided a better opportunity to observe the impact of model size in study 2. In total, there were 12 data generation conditions: 6 data generation models × 2 sample sizes.

**Table 5:** Study 2: Design factors for data generation

Population models	Number of latent factors	Number of items per latent factor	Number of item cross-loadings
Model C1 (F3-*I*10)	3	10	-
Model C2 (F6-*I*5)	6	5	-
Model C3 (F6-*I*10)	6	10	-
Model D1 (F3-*I*10-cld6)	3	10	6
Model D2 (F6-*I*10-cld6)	6	5	6
Model D3 (F6-*I*10-cld12)	6	10	12

Latent factor correlation and cross-loading magnitude fixed to 0.85 and 0.5, respectively

For model name, F = number of latent factors, *I* = number of items, and cld = number of cross-loadings. For example, “F3-*I*5” reads as three-factor model with five items, and “F6-*I*10-cld12” should be read as six-factor model with ten items and 12 cross-loadings

#### Population-level analysis

Population fit indices for both correctly specified and misspecified models were obtained as follows. For each of the models without cross-loadings, C1 to C3, a correctly specified model and two misspecified models were fitted. The number of latent factors was misspecified such that the model with three factors was misspecified as having two and then one factor, while the models with six factors were misspecified as four- and then as two-factor models. For each of the models with cross-loadings, D1 to D3, a correctly specified model and two misspecified models were examined. The cross-loadings were misspecified such that models with six cross-loadings were misspecified as having four and zero cross-loadings, while the model with 12 cross-loadings was misspecified as having eight and zero cross-loadings (see Table A2 in the Appendix for more design facet details). These designs enabled the assessment of the effects of high- and low-level misspecification in more complex models.

#### Sample-level analysis

The sample-level analysis designs were similar to the population-level analysis design described above, but we only analyzed the correctly specified model and the most severely misspecified models due to long execution time with large model sizes (see the models with bold fonts in Table 2 and Table A2 in the Appendix). The following priors were used for primary loadings in study 2: (1) Diffuse prior: *λ* ~ *N*(0, 1000), (2) AlignedSV: *λ* ~ *N*(${\mu }_{\lambda }$, 0.01), and (3) DivergentSV: *λ* ~ *N*(0.5, 0.01). AlignedLV and DivergentLV were shown to have had similar effects as Diffuse prior on all fit indices in study 1 and thus were not included here. Overall, a total of 72 conditions were analyzed: 12 analysis models × 2 sample sizes × 3 priors. The BSEM implementation and outcome evaluation followed the same criteria used in study 1. In addition, we used the fit indices’ 90% credible intervals to classify the models into poor, inconclusive, and good fit to the data.

### Results for study 2

#### Convergence rate

The convergence rate for study 2 was 100% or nearly 100% in most conditions. However, in some instances, AlignedSV and DivergentSV had convergence rates ranging from 71 to 100%, slightly lower than Diffuse prior (99% to 100%). One exception was DivergentSV in the most complex model with cross-loadings at *n* = 500 (model D3-1), where the convergence rate was merely 46% (see Table A3 in the Appendix).^18^

#### Analysis of variance

The ANOVA results for study 2 in Table 1 indicated that for Model C, prior type, latent factor misspecification, and their interaction had large effects on the estimated values of the fit indices. For model D, cross-loading misspecification, prior type, and sample size had moderate to large effect on all fit indices with some exceptions (see lower right section in Table 1). Other design factors, including the number of items, the number of latent factors, and number of cross-loadings, had negligible impact on the fit measures**.**

#### Fit value estimate

**Fig. 4:**
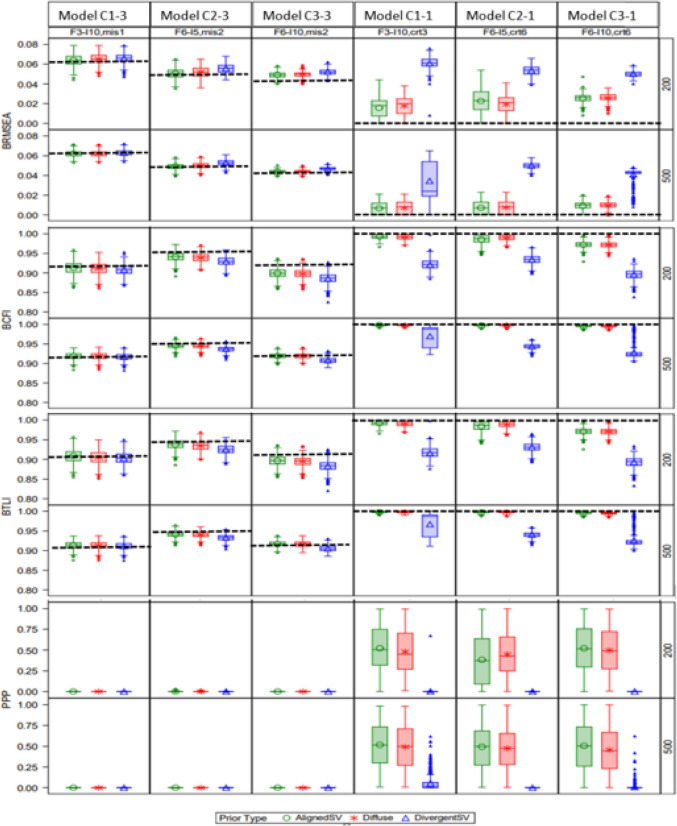
Study 2: Effect of prior specification, sample size, and model latent factor misspecification on BRMSEA, BCFI, and BTLI (with dotted lines representing population values). *Note:* First three columns contain misspecified models: C1-3 = F3-*I*10, mis1; C2-3 = F6-*I*5, mis2; C3-3 = F6-*I*10, mis2. Last three columns were correctly specified: C1-1 = F3-*I*10, crt3; C2-1 = F6-*I*5, crt6; C3-1 = F6-*I*10, crt6.. For model name, F = # of latent factors, *I* = # items, crt = correct specification, mis = misspecification. For example, “F3-*I*5*, crt3*” reads as a three-factor model with five items correctly specified as a three-factor model, and “F3-*I*5*, mis2*” reads as a three-factor model with five items misspecified as a two-factor model. Reference line is at the population value

**Fig. 5:**
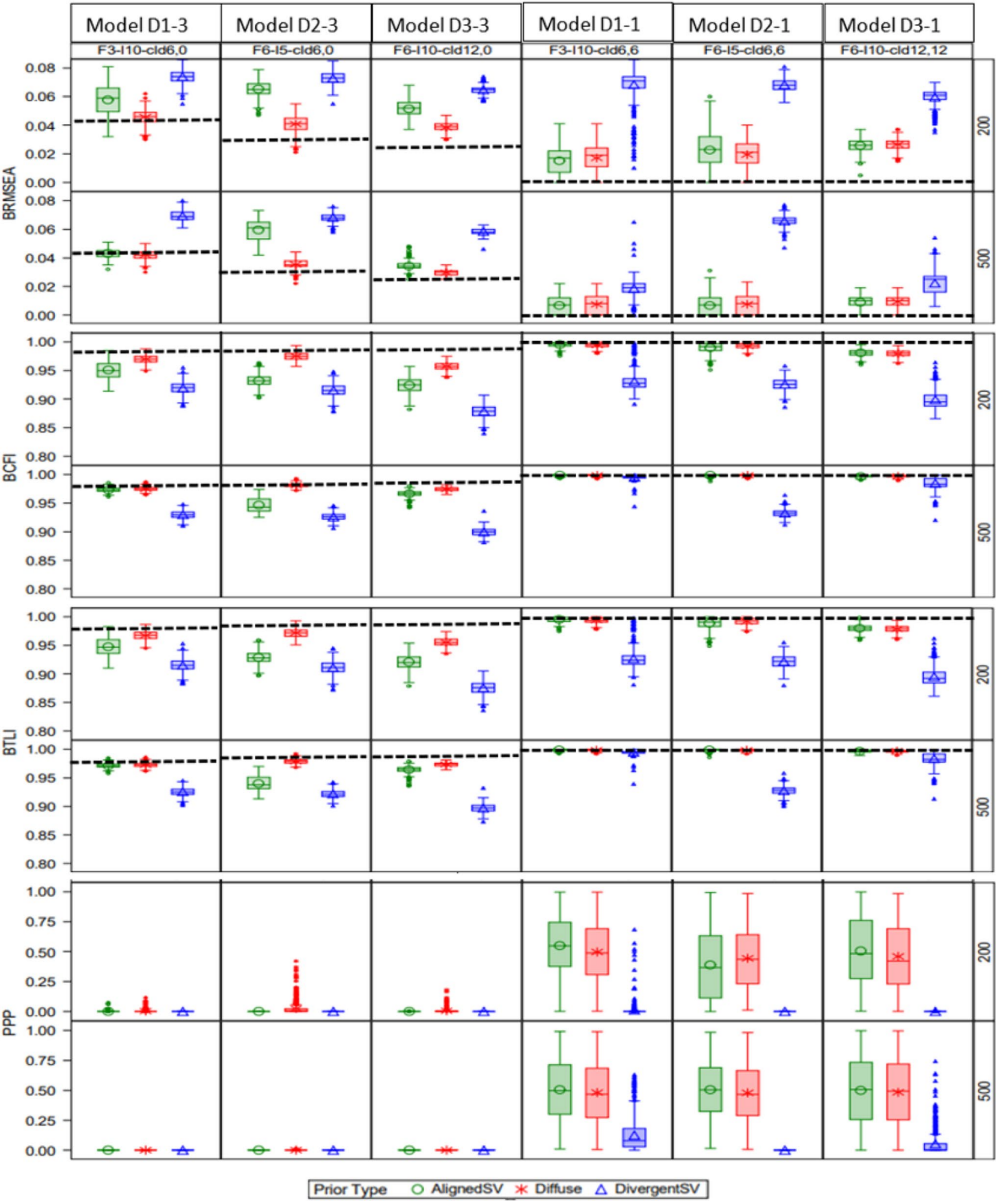
Study 2: Effect of prior specification, sample size, and cross-loading misspecification on BRMSEA, BCFI, and BTLI. Note: First three columns contain misspecified models: D1-3 = F3-I10-cld6,0; D2-3 = F6-I5-cld6,0; D3-3 = F6-I10-cld12,0. Last three columns were correctly specified: D1-1 = F3-I10-cld6,6; D2-1 = F6-I5-cld6,6; D3-1 = F6-I10-cld12,12. For model name, F = # of latent factors, I = # items, and cld = # of cross-loadings. For example, “F6-I10-cld12, 12” should be read as six-factor model with ten items and 12 cross-loadings specified as having 12 cross-loadings. Reference line is at the population value

Figure 4 shows the distributions of BRMSEA, BCFI, BTLI, and PP*p* with latent factor misspecification. For the correctly specified models (C1-1, C2-1, and C3-1), increasing model size led to slightly higher BRMSEA and lower BCFI and BTLI for AlignedSV and Diffuse priors, indicating a slight decline in model fit. PP*p* was less sensitive to model size, but it indicated poor model fit with DivergentSV even in correctly specified models. For misspecified models (C1-3, C2-3, and C3-3), increasing model size slightly enhanced the likelihood of signaling bad fit for models with misspecification. A similar pattern was observed for models with cross-loading misspecification (Fig. 5). The mean of all fit indices exhibited mostly negative bias compared with their population values when an informative but inaccurate prior (DivergentSV) was specified. The mean and standard deviation of the fit indices’ estimates are summarized in Tables A4 and A5 of the Appendix.

#### False and true positive rates

**Fig. 6:**
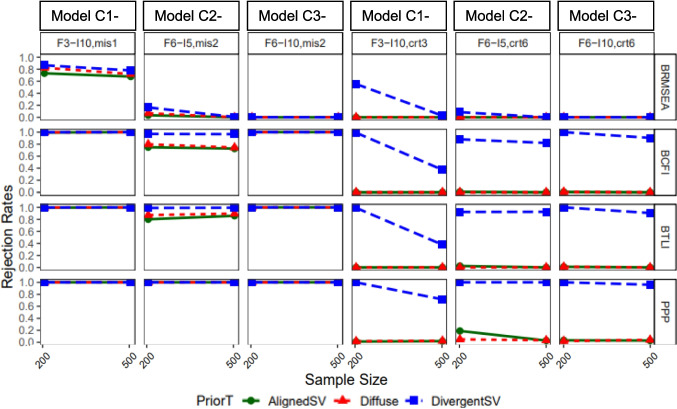
Study 2: Rejection rate across prior specification, sample size and model latent factor misspecification for BRMSEA, BCFI, BTLI, and PPp. *Note:* Latent factor correlation was fixed to 0.85 (informed by study 1). From left to right, columns 1–3 represent misspecified models with increasing levels of model complexity. Columns 4–6 are correctly specified models in the same order as columns 1–3. Models were named as follows: F = number of latent factors, *I* = number of items, crt = correct specification, mis = misspecification. For example, “F3-I10*,* crt3” reads as three-factor model with ten items correctly specified as a three-factor model, and “F6-I5, mis2” reads as six-factor model with five items misspecified as a two-factor model

Figure 6 presents the rejection rate for each fit index with varying levels of latent factor misspecifications. Overall, BRMSEA performed poorly in flagging model misspecification. Its power decreased as model size increased and ranged from about 80% to approximately 0% at the worst instance (see columns 1 to 3 of Fig. 6). Except for BRMSEA, all other fit indices yielded nearly 100% power across different model sizes (columns 1 to 3). The exception was in the condition where a six-factor model (model C2-3) was misspecified as having two factors; power of BCFI and BTLI was around 70–90% with AlignedSV and Diffuse priors. In terms of false positive rates, the fit indices prior performed well with AlignedSV and Diffuse, indicating an approximately 0% false positive rate across all model sizes. For DivergentSV, the false positive rate was inflated to about 50% for BRMSEA and worsened (ranging from about 80% to 100%) for BCFI, BTLI, and PP*p* as model size increased.

**Fig. 7:**
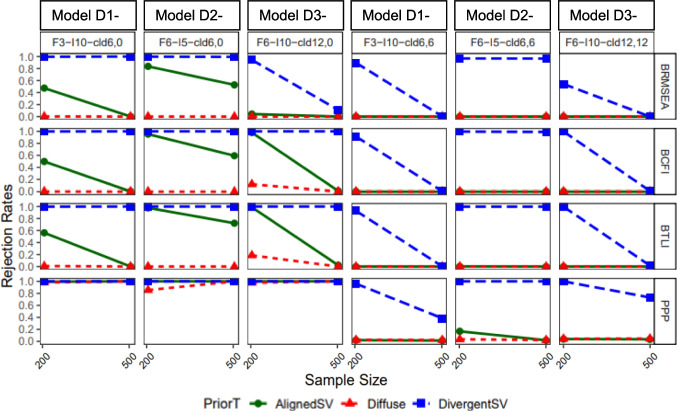
Study 2: Rejection rate across prior specification, sample size, and cross-loading misspecification for BRMSEA, BCFI, and BTLI, and PPp. *Note:* Latent factor correlation was fixed to 0.85 and cross-loading magnitude was fixed to 0.5 (informed by study 1). PP*p* values are produced in M*plus* only when priors are specified with small variance, hence the absence of Diffuse prior in the last row. From left to right, columns 1–3 represent misspecified models with increasing levels of model complexity. Columns 4–6 are correctly specified models in the same order as columns 1–3. Models were named as follows: F = number of latent factors, *I* = number of items, and cld = number of cross-loadings. For example, “F6-I10-cld12, 0” should be read as a six-factor model with ten items and 12 cross-loadings misspecified as having no cross-loadings. “F6-I10-cld12, 12” should read as a six-factor model with ten items and 12 cross-loadings correctly specified as having 12 cross-loadings

Figure 7 presents the rejection rates for models with varying levels of cross-loading misspecifications. Model size seems not to have had a consistent impact on true and false positive rates. With respect to true positive rate, PP*p* performed relatively well with all prior types indicating about 100% power. But for BRMSEA, BCFI, and BTLI, AlignedSV indicated decreased power as sample size increased (from *n* = 200 to 500), while Diffuse prior showed nearly no power to detect cross-loading misspecification. DivergentSV on the other hand indicated about 100% power for all fit indices except for BRMSEA, with the most complex model having 12 item cross-loadings, misspecified as zero cross-loadings (model D3-3). For false positive rate, all fit indices indicated an approximately 0% rejection rate when the Aligned and Diffuse prior was specified, but DivergentSV performed poorly, indicating a close to 100% false positive rate across all fit indices at *n* = 200.

#### Evaluating BCFI, BTLI, and BRMSEA with 90% credible interval

The performance of BCFI, BTLI, and BRMSEA was assessed using the 90% credible interval. Model fit to data was described as *good* if the lower bound for BCFI and/or BTLI is greater than 0.95 or if upper bound for BRMSEA is less than 0.06. Model fit to data was described as *poor* if the upper bound of BCFI and/or BTLI is less than 0.95 or the lower bound for BRMSEA is greater than 0.06. Lastly, model fit to data was said to be *inconclusive* when the upper and lower bound for BCFI or BTLI includes 0.95 or when the lower and upper bound of BRMSEA is inclusive of 0.06.

**Fig. 8:**
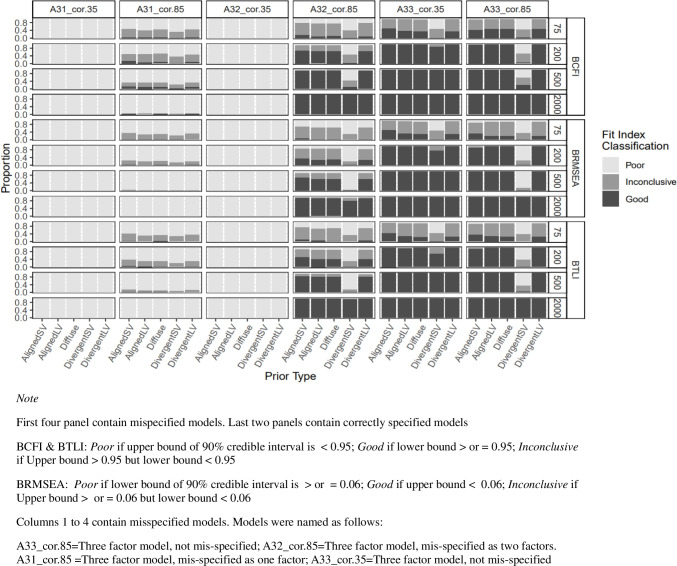
Study 1: Classification of BCFI, BRMSEA, and BTLI based on 90% credible interval for models with latent factor misspecification

**Fig. 9:**
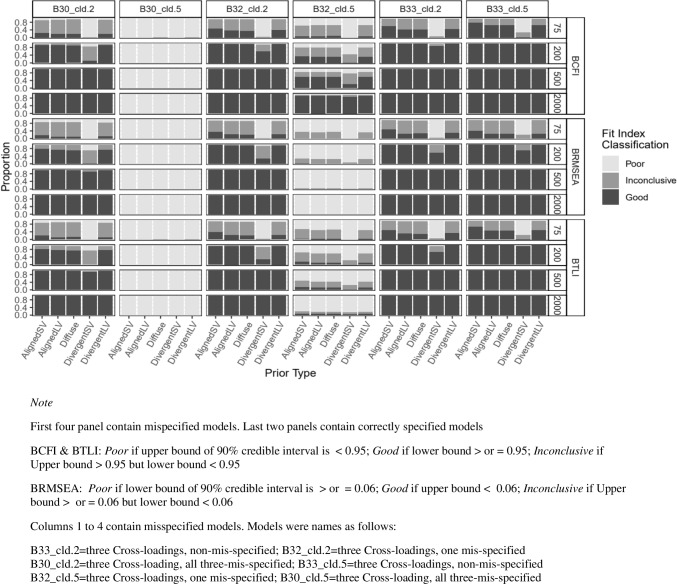
Study 1: Classification of BCFI, BRMSEA, and BTLI based on 90% credible interval for models with cross-loading misspecification

**Fig. 10:**
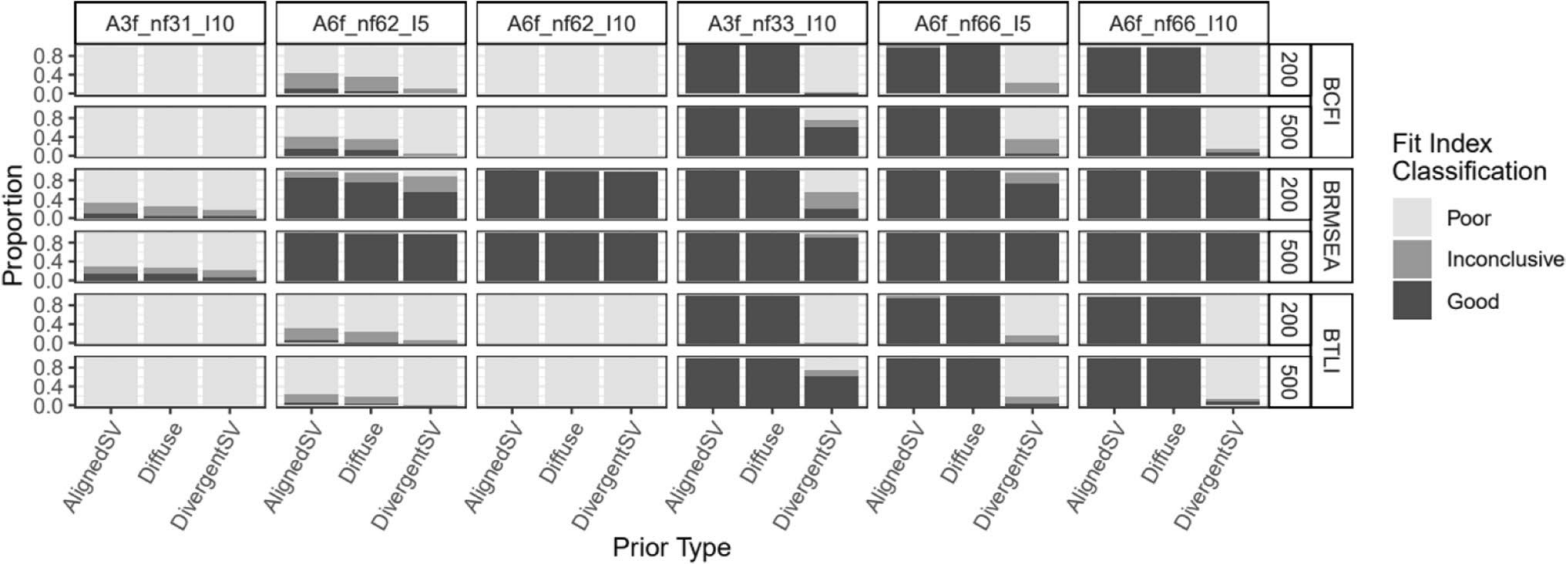
Study 2: Classification of BCFI, BRMSEA, and BTLI based on 90% credible interval for models with latent factor misspecification. *Note:* First three panels contain misspecified models. Last three panels contain correctly specified models. BCFI & BTLI: *Poor* if upper bound of 90% credible interval is < 0.95; *good* if lower bound ≥ 0.95; *inconclusive* if upper bound ≥ 0.95 but lower bound < 0.95. BRMSEA: *Poor* if lower bound of 90% credible interval is ≥ 0.06; *good* if upper bound < 0.06; *inconclusive* if upper bound ≥ 0.06 but lower bound < 0.06. Models were named as follows: nf = number of latent factors, *I* = number of items. A3f_nf31_*I*10: three-factor model with ten items each, misspecified as one-factor model. A3f_nf33_*I*10: three-factor model with ten items each, correctly specified as a three-factor model. A6f_nf62_*I*5: six-factor model with five items each, misspecified as a two-factor model. A6f_nf66_*I*5: six-factor model with five items each, correctly specified as a six-factor model, etc

**Fig. 11:**
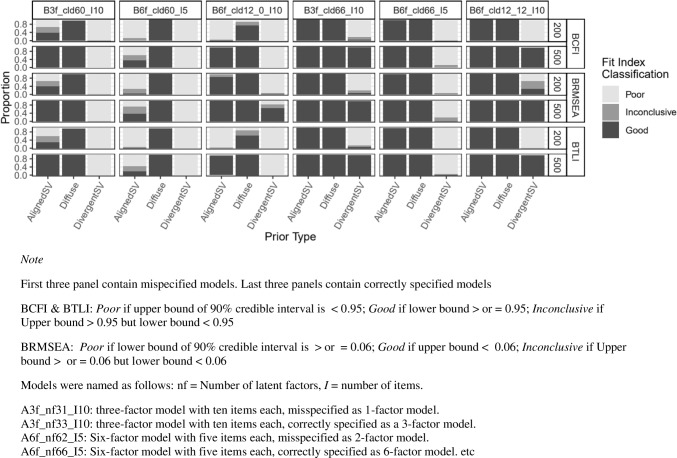
Study 2: Classification of BCFI, BRMSEA, and BTLI based on 90% credible interval for models with cross-loading misspecification

For misspecified models in study 1, BCFI, BTLI, and BRMSEA clearly indicated poor model fit for more severely misspecified models (e.g., panels 1 and 3 in Fig. 8 and panel 2 in Fig. 9). However, as the model size and complexity increased (study 2), all three fit indices were less likely to signal poor model fit for misspecified models, with BRMSEA performing worse in this direction. At the instance of the worst performance with more complex models, BRMSEA indicated near 100% good model fit for misspecified model dimensionality for all prior types (see panels 2 and 3 in Fig. 10). When cross-loadings were ignored, BTLI and BCFI also indicated 100% good model fit but only when Diffuse prior was specified (see first three panels in Fig. 11).

For the correctly specified models, in study 1, BCFI, BTLI, and BRMSEA all signaled poor or inconclusive fit for correctly specified models about 50% of the time for the smallest sample size (*n* = 75), irrespective of the type of misspecification (latent factor or cross-loadings), except for DivergentSV which indicated inconclusive or poor fit most of the time (see last two panels in Figs. 8 and 9). However, this trend decreased drastically. As sample size increased from 200 to 500, all priors, except for DivergentSV, indicated good model fit for correctly specified models. With DivergentSV, all three fit indices consistently indicated poor or inconclusive model fit in conditions with correctly specified factor dimension when factor correlations were 0.85 (last panel of Fig. 8). However, when sample increased to 2000, all fit indices indicated good model fit for correctly specified models. As model complexity increased, all fit indices indicated good fit for models assigned AlignedSV and Diffuse priors. Overall, with large model size (study 2), DivergentSV was associated with inconclusive or poor model fit in more than half of the conditions (Figs. 10 and 11).

### Summary of findings for study 2

For latent factor misspecifications, increasing model size slightly reduced the power of BCFI, BTLI, and PPp, while BRMSEA’s power declined rapidly to near zero. For cross-loading misspecifications, power generally decreased, varying by prior types, with PP*p* showing the highest stability and power. BRMSEA, BCFI, and BTLI had near-zero power for larger models with Diffuse priors, whereas AlignedSV showed increased power for smaller samples. DivergentSV's type I error rates were inflated to nearly 1 in half of the conditions as model size increased, even with large samples.

## Discussion

Experimental psychology often deals with latent construct like memory, perception, or personality, which are not directly observable. BSEM allows for more nuanced modeling of these constructs by incorporating prior information and addressing model uncertainty, enabling researchers to test complex hypotheses about psychological processes. Evaluating the performance of BSEM fit indices contributes to both methodological advancements and the practical application of robust statistical models in experimental psychology. Understanding the extent of responsiveness of BSEM fit indices to priors helps ensure that the added benefits of using priors are appropriately reflected in the analysis. This in turn ultimately contributes to improving the reliability and interpretability of psychological research findings.

In this paper, we investigated prior sensitivity of Bayesian SEM fit indices (BRMSEA, BCFI, BTLI, and PP*p*) to latent factor and cross-loading misspecifications, with a focus on the impact of model size and prior sensitivity of these BSEM fit indices. BRMSEA, BCFI, BTLI, and PP*p* were examined using ANOVA, the mean and standard deviation of the fit index values, rejection rates (false and true positive rates), and credible intervals. A summary of key findings is presented below.

At the population level, model fit worsened as misspecification became more severe. However, the sample level analysis indicated that for study 1, the average fit indices values for BRMSEA, BCFI and BTLI were negatively biased, relative to their population values, when the sample size was small (*n* < 500). The greatest bias occurred when an informative but inaccurate prior (DivergentSV) was specified. This is consistent with previous research (Edwards & Konold, 2022; Hoofs et al., 2018; Winter & Depaoli, 2022b). For study 2, slightly larger BRMSEA and lower BCFI and BTLI were associated with increased model size.

When the number of latent factors were misspecified in study 1, all fit indices performed well with low latent factor correlation (0.35), but poorly under less severe latent factor misspecification (factor correlation of 0.85). For cross-loading misspecification, excluding PP*p*, power for BRMSEA, BCFI, and BTLI decreased with lower cross-loading magnitude and larger sample size across all prior types. When *n* $\le$ 200, the false positive rate increased to about 20% for all fit measures except DivergentSV, which inflated the false positive rates to about 80% or more. The less optimal performance of BSEM fit measures with small sample size aligns with their intended use for evaluating models with large sample sizes (*n* $\ge 1000$; Hoofs et al., 2018; Garnier-Villarreal & Jorgensen, 2020). However, even with large sample sizes, their overall performance was suboptimal under less severe misspecification. For study 2, the performance of BRMSEA, BTLI, and BCFI slightly declined with increased model size. BRMSEA performed the least effective in flagging model misspecification, while PP*p* was the most stable across all prior types.

Evaluation based on the 90% credible interval aligns with the overall findings: severely misspecified models were generally flagged as having poor fit, while less severe misspecifications showed lack of power, especially as model complexity increased. BRMSEA had the poorest performance, particularly with increased complexity. False positive rates rose significantly with DivergentSV compared to other priors, likely due to its inherent misalignment. Overall, BTLI and BCFI performed better than BRMSEA.

## Conclusions and implications

For less complex models (having about three latent factors, each with five indicators), (i) when latent factors or item cross-loadings were severely misspecified, with factor correlation $\le$ 0.35 or item cross-loadings magnitude $\ge$ 0.5, all fit indices performed well; (ii) irrespective of the prior type specified, BRMSEA, BTLI, and BCFI performed poorly when there was less severe misspecification and when *n* $\le 200$; (iii) when DivergentSV (informative but inaccurate prior) was specified, all fit indices yielded the greatest bias, lowest power, and inflated false positive rates. For more complex models (study 2), (iv) increasing model size reduced sensitivity for BCFI, BTLI, and BRMSEA*.* BRMSEA showed the worst performance, with decreasing power as model size increased, failing to reject misspecified models at instances, irrespective of prior types; (v) PP*p* demonstrated the most consistent performance across model sizes, prior types, and levels of misspecification.

Findings from this study suggest that empirical researchers using BRMSEA, BCFI, BTLI, and PP*p* to assess global fit of BSEM models should opt for highly informative priors only when there is certainty about prior knowledge. In situations where prior information is unclear, using noninformative prior is recommended, as they ensure reduced false positive rates, albeit with reduced power. As model size increases, using PP*p* is generally recommended over BCFI and BTLI given its balanced performance between false positive rate and power. BRMSEA is less reliable than all other fit measured considered and should receive the least consideration. Lastly, model fit should be interpreted cautiously when sample size is large ($\ge$ 2000) and there is less severe misspecification (e.g., large latent factor correlation, $\ge$ 0.85, and small cross-loading magnitude, $\le 0.2$). All fit indices tend to perform poorly in signaling poor fit for misspecified models in such instances.

## Limitations and future research

Findings from this study are limited by the number and combinations of conditions explored. Correlated errors among items were not investigated. Potentially, models with correlated error structures and different combinations of conditions other than those explored in this study may produce different outcomes. Evaluating prior sensitivity of BSEM fit indices under different combinations of conditions beyond this study will deepen knowledge in this respect. Comparative fit indices like BCFI and BTLI are based on incremental performance of a hypothesized model over a null or independent model. It is worth noting that the idea of a null model within the Bayesian framework that subsumes the presence of prior information is counter-intuitive (Hoofs et al., 2018). This poses a fundamental problem in conceptualizing a comparative fit index for BSEM models. While the use of noninformative priors may present some justification (Garnier-Villarreal & Jorgensen, 2020), further research is needed to fully address this concern, especially in the case of an informative prior.

## Supplementary Information

Below is the link to the electronic supplementary material.

Supplementary file1 (DOCX 360 KB)

## Data Availability

Computer-simulated data were analyzed, as described in the Method section. This study was not preregistered.

## Footnotes

1 What is considered a small variance could differ among researchers, but generally a normal prior with variance smaller than 1 is seen as a small-variance prior (Asparouhov & Muthén, 2017). Note that the degree of informativeness is always in relation to the data under consideration. Overall, as sample size increases, the data becomes more influential, and the impact of the prior on the posterior distribution diminishes.

2 Generally, priors that integrate to 1, hence a formal probability distribution, are referred to as proper prior, and those that do not integrate to 1 are referred to as improper prior (Gelman, 2006; van Erp et al., 2018). In this respect, all five priors used in this paper are proper priors. However, more specifically, comparing the naming convention for proper priors used in this paper with other papers: accurate and informative prior has been referred to as tight prior (Depaoli, 2012), and conservative or weakly informative prior (Hoofs et al., 2018), while an accurate but uninformative prior has been referred to as “weak” prior (Depaoli, 2012). Diffuse and noninformative prior has been referred to as “naïve” prior (Finch & Miller, 2019), and vague proper prior (van Erp et al., 2018).

3 These terms were used in Winter and Depaoli (2022b) in a similar sense as used in this paper.

4 Wu et al. (2014) presented a scenario where numerical evidence alone would not have been adequate in determining model fit to data (Fig. 1, p. 266). Reproduced here (see Figure A3 in the appendix).

5 Note that PPMC procedures could be designed to assess fit on specific part of a model, just as it could be designed for global model fit assessment.

6 Sufficient statistics are excluded because they summarize data based on the model’s parameters and, as a result, may not fully capture discrepancies between the model and the data.

7 Two of the most commonly used Bayesian analysis software packages, blavaan package in R (Merkle & Rosseel, 2018) and M*plus* (Muthén & Muthén, 1998–2023), utilized the chi-square discrepancy function in their implementation of PP*p.*

8 BRMSEA^DevM^ and BRMSEA^ppmc^ were the naming convention introduced by Garnier-Villarreal and Jorgensen (2020) to differentiate their version of BRMSEA from the version by Hoofs et al. (2018).

9 In the numerator, model complexity is represented by $df$, while ${\chi }^{2}$ captures model misfit.

10 BRMSEA^DevM^, BCFI, and BTLI were adopted for implementation in Mplus 8, (Asparouhov & Muthén, 2020) and that is the version of the fit indices evaluated in this paper.

11 Hoofs et al. (2018) observed that the idea of a null model in the presence of prior information is contradictory. Garnier-Villarreal and Jorgensen (2020) justified their proposal for incremental fit indices like BTLI and BCFI with a null model on the grounds that their proposal considered only the case of noninformative prior.

12 The potential impact on computing BSEM fit indices when *pD* is less than *q* has not yet been directly studied.

13 Also referred to as posterior probability intervals (PPI; Van de Schoot et al., 2014).

14 What the authors referenced as inaccurate prior is a prior specified with small variance (0.005) but with factor loading and factor covariance having values different from their population mean and covariance, respectively. In this paper’s naming convention, this prior belongs to the DivergentSV (divergent prior with small variance) category.

15 *δ* ~ *IG*(− 1, 0): inverse gamma prior, Φ ~ *IW*(0, − 7): inverse Wishart prior.

16 M*plus* 8 defines PSR < *a* for each parameter as convergence criterion, where *a* = 1 + BCONVERGENCE * *f*. The multiplicity factor (*f*) takes the value 1 when there is just one parameter in the model. But *f* = 2 when there are two or more parameters in the model. For a large model with the default BCONVERGENCE = 0.05, *a* = 1 + (0.05 *2). This yields PSR < 1.10. (Muthén & Muthén, 2017, p. 704; Asparouhov & Muthén, 2023).

17 The total number of replications needed to obtain the required 500 converged solutions for each condition differed. For all conditions, an initial run of 500 replications was first implemented. If all 500 replications converged, convergence rate was 100%. This means that for the condition with convergence rate of 66%, 330 replications out of the first 500 replications have converged. By extension, a minimum of 758 replications will be needed to obtain 500 converged replications for this condition.

18 More replications run was used to realize the total of 500 converged solutions. For example, for the condition with the lowest convergence rate (46%), only 230 replications out of the first 500 replications converged. By extension, a minimum of 1087 replications will be needed to obtain 500 converged solutions for this condition.

## Code availability

Data generation and analysis code are available at: https://osf.io/yjgua/?view_only=affdbf4d5858444fada94c6f3565210e
