# Correction: Probing beyond: The impact of model size and prior informativeness on Bayesian SEM fit indices

**DOI:** 10.3758/s13428-025-02672-9

**Published:** 2025-04-16

**Authors:** Ejike Edeh, Xinya Liang, Chunhua Cao

**Affiliations:** 1 Department of Counseling, Leadership, and Research Methods, University of Arkansas, 751 W. Maple St, Fayetteville, AR 72701 USA; 2 Educational Studies, College of Education, University of Alabama, 316 A Carmichael Hall, Tuscaloosa, AL USA

**Correction: Behavior Research Methods (2025) 57:108**

10.3758/s13428-025-02609-2

The original online version of this article was revised.

This abstract: Examining how well a hypothesized model fits observed data is pivotal in Bayesian structural equation modeling (SEM). Recent efforts have aimed to formulate Bayesian SEM fit indices akin to those used in frequentist SEM. However, the assessment of these fit indices in Bayesian SEM has been limited in scope. This research entailed two simulation studies to explore the impact of various factors, including prior choices, model misspecification, model size, and sample size on the sensitivity of fit indices. The first study investigated the prior sensitivity of Bayesian SEM fit indices (BRMSEA, BCFI, BTLI, and PPp) under latent factor and cross-loading misspecifications. The second study explored how alterations in model complexity influenced the prior sensitivity of these fit indices to model misspecifications. Findings indicated a robust performance of Bayesian fit indices under relatively severe model misspecification, suggesting a preference for highly informative priors when prior knowledge was certain. As model size increased, PPp exhibited a balanced performance between true and false positive rates, whereas BRMSEA was less reliable in the presence of latent factor misspecification. Implications for applied research were discussed.

Was inadvertently left out of the final version.

And the Key words: BSEM, Bayesian fit indices, model size, prior sensitivity, were also left out of the article when it was published.

