# Supplementary material for: Probing beyond: The impact of model size and prior informativeness on Bayesian SEM fit indices
Source: Behav Res Methods. 2025 Mar 5;57(4):108. doi: 10.3758/s13428-025-02609-2 (PMC11882663; doi:10.3758/s13428-025-02609-2)
Supplement: Supplementary file 1 — Supplementary file1 (DOCX 360 KB) [file 13428_2025_2609_MOESM1_ESM.docx]

**Appendix: Tables**

| **Table A1**  *Study 1: Convergence Rate* | | | | | | | | | | | | | | |
| --- | --- | --- | --- | --- | --- | --- | --- | --- | --- | --- | --- | --- | --- | --- |
|  | **Models** | **A3** | **A3** | **A2** | **A2** | **A1** | **A1** |  | **B3** | **B3** | **B2** | **B2** | **B1** | **B1** |
|  |  | **(A31**  **cor.35)** | **(A31**  **cor.85)** | **(A32**  **cor.35)** | **(A32**  **cor.85)** | **A33**  **cor.35** | **A33**  **cor.85** |  | **B30**  **cld.2** | **B30**  **cld.5** | **B32**  **cld.2** | **B32**  **cld.5** | **B33**  **cld.2** | **B33**  **cld.5** |
| **N** | **Prior Type** |  |  |  |  |  |  |  |  |  |  |  |  |  |
| 75 | AlignedLV | 1 | 1 | .98 | 1 | 1 | 1 |  | 1 | 1 | 1 | 1 | 1 | 1 |
|  | AlignedSV | 1 | 1 | 1 | 1 | 1 | .88 |  | .99 | .87 | 1 | 1 | 1 | 1 |
|  | Diffuse | 1 | 1 | .98 | 1 | 1 | 1 |  | 1 | 1 | 1 | 1 | 1 | 1 |
|  | DivergentLV | 1 | 1 | .99 | 1 | 1 | 1 |  | 1 | 1 | 1 | 1 | 1 | 1 |
|  | DivergentSV | 1 | 1 | 1 | 1 | .82 | 1 |  | .84 | .97 | .87 | .98 | .79 | .77 |
|  |  |  |  |  |  |  |  |  |  |  |  |  |  |  |
| 200 | AlignedLV | 1 | 1 | .97 | 1 | 1 | 1 |  | 1 | 1 | 1 | 1 | 1 | 1 |
|  | AlignedSV | 1 | 1 | 1 | 1 | 1 | .97 |  | 1 | 1 | 1 | 1 | 1 | 1 |
|  | Diffuse | 1 | 1 | .97 | 1 | 1 | 1 |  | 1 | 1 | 1 | 1 | 1 | 1 |
|  | DivergentLV | 1 | 1 | .97 | 1 | 1 | 1 |  | 1 | 1 | 1 | 1 | 1 | 1 |
|  | DivergentSV | 1 | 1 | 1 | 1 | 1 | 1 |  | .94 | .74 | .99 | .99 | .99 | 1 |
|  |  |  |  |  |  |  |  |  |  |  |  |  |  |  |
| 500 | AlignedLV | 1 | 1 | .91 | 1 | 1 | 1 |  | 1 | 1 | 1 | 1 | 1 | 1 |
|  | AlignedSV | 1 | 1 | .99 | 1 | 1 | 1 |  | 1 | 1 | 1 | 1 | 1 | 1 |
|  | Diffuse | 1 | 1 | .90 | 1 | 1 | 1 |  | 1 | 1 | 1 | 1 | 1 | 1 |
|  | DivergentLV | 1 | 1 | .91 | 1 | 1 | 1 |  | 1 | 1 | 1 | 1 | 1 | 1 |
|  | DivergentSV | 1 | 1 | .97 | 1 | 1 | 1 |  | 1 | 1 | 1 | 1 | 1 | 1 |
|  |  |  |  |  |  |  |  |  |  |  |  |  |  |  |
| 2000 | AlignedLV | 1 | 1 | **.66** | 1 | 1 | 1 |  | 1 | 1 | 1 | 1 | 1 | 1 |
|  | AlignedSV | 1 | 1 | **.72** | 1 | 1 | 1 |  | 1 | 1 | 1 | 1 | 1 | 1 |
|  | Diffuse | 1 | 1 | **.66** | 1 | 1 | 1 |  | 1 | 1 | 1 | 1 | 1 | 1 |
|  | DivergentLV | 1 | 1 | **.66** | 1 | 1 | 1 |  | 1 | 1 | 1 | 1 | 1 | 1 |
|  | DivergentSV | 1 | 1 | **.70** | 1 | 1 | 1 |  | 1 | 1 | 1 | 1 | 1 | 1 |
| *Note:* cor = correlation and cld=cross-loading*;* A33_cor.35 / ( _cor.85) =Three factor model, correctly specified with factor correlation varied at 0.35 and 0.85; A32_cor.35 / ( _cor.85) =Three factor model, mis specified as two factors with factor correlation varied at 0.35 and 0.85; A31_cor.35 / ( _cor.85) = Three factor model, mis specified as 1 factor with factor correlation varied at 0.35 and 0.85; B33_cld.2 / (_cld.5)=three Cross-loadings, correctly specified with cross-loading magnitude varied at .2 and .5; B32_cld.2 / (_cld.5) =three Cross-loadings, mis-specified by ignoring one, with cross-loading magnitude varied at .2 and .5; B30_cld.2 / (_cld.5) =three Cross-loadings, mis-specified by ignoring all three, with cross-loading magnitude varied at .2 and .5; | | | | | | | | | | | | | | |

| **Table A2**  *Simulation design for data analysis models for Studies 1 and 2* | | | | | |
| --- | --- | --- | --- | --- | --- |
| *Study 1* | Number of latent factors in population model | Number of latent factors specified | Number of items per latent factor | Number of item cross loadings in population model | Number of item cross loadings specified |
| *Model A*: | | | | |  |
| A1 (A33_cor.35 / .85) | 3 | 3 | 5 | - | - |
| A2 (A32_cor.35 / .85) | 3 | 2 | 5 | - | - |
| A3 (A31_cor.35 / .85) | 3 | 1 | 5 | - | - |
| *Model B* | | | | |  |
| B1 (B33_cld.2 /.5) | 3 | 3 | 5 | 3 | 3 |
| B2 (B32_cld.2 /.5) | 3 | 2 | 5 | 3 | 2 |
| B3 (B30_cld.2 /.5) | 3 | 0 | 5 | 3 | 0 |
|  |  |  |  |  |  |
| *Study 2* |  |  |  |  |  |
| *Model C* |  |  |  |  |  |
| **C1-1 (F*3-I*10*, crt3*)** | 3 | 3 | 10 | - | - |
| C1-2 (F3-*I*10*, mis2*) | **3** | **2** | **10** | **-** | **-** |
| **C1-3 (F3*-I*10*, mis1*)** | 3 | 1 | 10 | - | - |
| **C2-1 (F6*-I*5*, crt6*)** | 6 | 6 | 5 | - | - |
| C2-2 (F6-*I*5*, mis4*) | **6** | **4** | **5** | **-** | **-** |
| **C2-3 (F6-*I*5*, mis2*)** | 6 | 2 | 5 | - | - |
| **C3-1 (F6-*I*10*, crt6*)** | 6 | 6 | 10 | - | - |
| C3-2 (F6-*I*10*, mis4*) | **6** | **4** | **10** | **-** | **-** |
| **C3-3 (F6-*I*10*, mis2*)** | 6 | 2 | 10 | - | - |
|  |  |  |  |  |  |
| *Model D* |  |  |  |  |  |
| **D1-1 (F3-*I*10-cld6, 6)** | 3 | 3 | 10 | 6 | 6 |
| D1-2 (F3-*I*10-cld6, 4) | **3** | **3** | **10** | **6** | **4** |
| **D1-3 (F3-*I*10-cld6, 0)** | 3 | 3 | 10 | 6 | 0 |
| **D2-1 (F6-*I*5-cld6, 6)** | 6 | 6 | 5 | 6 | 6 |
| D2-2 (F6-*I*5-cld6, 4) | **6** | **6** | **5** | **6** | **4** |
| **D2-3 (F6-*I*5-cld6, 0)** | 6 | 6 | 5 | 6 | 0 |
| **D3-1 (F6-*I*10-cld12, 12)** | 6 | 6 | 10 | 12 | 12 |
| D3-2 (F6-*I*10-cld12, 8) | **6** | **6** | **10** | **12** | **8** |
| **D3-3 (F6-*I*10-cld12, 0**) | 6 | 6 | 10 | 12 | 0 |
| ***Note:*** models were named as follows: *c*or = correlation and cld=cross-loading*;* A33_cor.35 / cor.85) =Three factor model, correctly specified with factor correlation varied at 0.35 and 0.85; A32_cor.35 / cor.85) =Three factor model, mis specified as two factors with factor correlation varied at 0.35 and 0.85; A31_cor.35 / cor.85) = Three factor model, mis specified as 1 factor with factor correlation varied at 0.35 and 0.85; B33_cld.2 / cld.5)=three Cross-loadings, correctly specified with cross-loading magnitude varied at .2 and .5; B32_cld.2 / cld.5) =three Cross-loadings, mis-specified by ignoring one, with cross-loading magnitude varied at .2 and .5; B30_cld.2 / cld.5) =three Cross-loadings, mis-specified by ignoring all three, with cross-loading magnitude varied at .2 and .5;  ***For study 2****,* models were named as follows: F = Number of latent factors, *I* = number of items, crt = correct specification, mis=misspecification, and cld = number of cross-loading. For example, “F3-*I*5*, crt3*” reads as: three-factor model with five items correctly specified as a three-factor model., and “F3-*I*5*, mis2*” reads: three-factor model with five items misspecified as a two-factor model. Similarly, “F6-*I*10-cld12, 8” should be read as: Six-factor model with ten items and 12 cross-loadings misspecied as having eight cross-loadings | | | | | |

| **Table A3**  *Study 2: Convergence Rate* | | | | | | | | | | | | | | | |
| --- | --- | --- | --- | --- | --- | --- | --- | --- | --- | --- | --- | --- | --- | --- | --- |
|  | **Models** | **C1-1** | **C1-3** | **C2-1** | | **C2-3** | **C3-1** | **C3-3** |  | **D1-1** | **D1-3** | **D2-1** | **D2-3** | **D3-1** | **D3-3** |
| **N** | **Prior Type** |  |  |  |  | |  |  |  |  |  |  |  |  |  |
| 200 | AlignedSV | 1 | 1 | .71 | 1 | | .96 | 1 |  | 1 | .88 | .77 | .97 | .98 | .73 |
|  | Diffuse | 1 | 1 | 1 | 1 | | 1 | 1 |  | 1 | 1 | 1 | .99 | 1 | 1 |
|  | DivergentSV | .98 | 1 | 1 | 1 | | 1 | 1 |  | .90 | 1 | 1 | 1 | .83 | 1 |
|  |  |  |  |  | |  |  |  |  |  |  |  |  |  |  |
|  |  |  |  |  | |  |  |  |  |  |  |  |  |  |  |
| 500 | AlignedSV | 1 | 1 | 1 | | 1 | 1 | 1 |  | 1 | .99 | 1 | .86 | 1 | .73 |
|  | Diffuse | 1 | 1 | 1 | | 1 | 1 | 1 |  | 1 | 1 | 1 | 1 | 1 | 1 |
|  | DivergentSV | .92 | 1 | 1 | | 1 | .73 | 1 |  | 1 | 1 | .98 | 1 | **.46** | 1 |
| Note:  C1-1= F3-*I*10, crt3; C1-3 = F3-*I*10, mis1; C2-1 = F6-*I*5, crt6; C2-3 = F6-*I*5, mis2; C3-1 = F6-*I*10, crt6; C3-3 = F6-*I*10, mis2;  D1-1= F3-*I*10-cld6,6; D1-3 = F3-*I*10-cld6,0; D2-1 = F6-*I*5-cld6,6; D2-3 = F6-*I*5-cld6,0; D3-1 = F6-*I*10-cld12,12;  D3-3 = F6-*I*10-cld12,0  For model name, F = # of latent factors, *I* = # items, crt = correct specification, mis=misspecification, and cld =# of cross-loading. For example, “F3-*I*5*, crt3*” reads as: three-factor model with five items correctly specified as a three-factor model., and “F3-*I*5*, mis2*” reads: three-factor model with five items misspecified as a two-factor model. Similarly, “F6-*I*10-cld12, 12” should be read as: Six-factor model with ten items and 12 cross-loadings specified as having 12 cross-loadings. | | | | | | | | | | | | | | | |

| **Table A4**  *Study 2: Mean (SD) of BRMSEA, BCFI, and BTLI and PPp by sample size, prior specification, covariance structure and latent factor misspecification.* | | | | | | | | | | |
| --- | --- | --- | --- | --- | --- | --- | --- | --- | --- | --- |
|  |  |  | **Three factors:**  **Mis-specified as one**  **and Correct** | | **Six factors:**  **Mis-specified as two**  **and Correct** | | |  | **Six factors:**  **Mis-specified as two**  **and Correct** | |
|  |  | **Models** | **C1-3** | **C1-1** |  | **C2-3** | **C2-1** |  | **C3-3** | **C3-1** |
|  | **n** | **Prior Type** | **F3-I10, mis1** | **F3-I10, crt3** |  | **F6-I5, mis2** | **F6-I5, crt6** |  | **F6-I10, mis2** | **F6-I10, crt6** |
| **Population BRMSEA** | |  | **0.062** | **0.000** |  | **0.049** | **0.000** |  | **0.043** | **0.000** |
|  | 200 | DivergentSV | 0.07 (0.01) | 0.06 (0.01) |  | 0.06 (0.00) | 0.05 (0.01) |  | 0.05 (0.00) | 0.05 (0.00) |
|  |  | Diffuse & AlignedSV | 0.06 (0.01) | 0.02 (0.01) |  | 0.05 (0.01) | 0.02 (0.01) |  | 0.05 (0.00) | 0.03 (0.00) |
|  |  |  |  |  |  |  |  |  |  |  |
|  | 500 | DivergentSV | 0.06 (0.00) | 0.03 (0.02) |  | 0.05 (0.00) | 0.05 (0.00) |  | 0.05 (0.00) | 0.04 (0.01) |
|  |  | Diffuse & AlignedSV | 0.06 (0.00) | 0.01 (0.01) |  | 0.05 (0.00) | 0.01 (0.00) |  | 0.04 (0.00) | 0.01 (0.00) |
| **Population BCFI** | |  | **0.920** | **1.000** |  | **0.947** | **1.000** |  | **0.923** | **1.000** |
|  | 200 | DivergentSV | 0.91 (0.01) | 0.92 (0.01) |  | 0.93 (0.01) | 0.93 (0.01) |  | 0.89 (0.01) | 0.90 (0.01) |
|  |  | Diffuse & AlignedSV | 0.91 (0.02) | 0.99 (0.01) |  | 0.94 (0.01) | 0.99 (0.01) |  | 0.90 (0.01) | 0.97 (0.01) |
|  |  |  |  |  |  |  |  |  |  |  |
|  | 500 | DivergentSV | 0.92 (0.01) | 0.97 (0.03) |  | 0.94 (0.01) | 0.94 (0.01) |  | 0.91 (0.01) | 0.93 (0.02) |
|  |  | Diffuse & AlignedSV | 0.92 (0.01) | 1.00 (0.00) |  | 0.95 (0.01) | 1.00 (0.00) |  | 0.92 (0.01) | 1.00 (0.00) |
| **Population BTLI** | |  | **0.914** | **1.000** |  | **0.943** | **1.000** |  | **0.921** | **1.000** |
|  | 200 | DivergentSV | 0.90 (0.02) | 0.92 (0.01) |  | 0.93 (0.01) | 0.93 (0.01) |  | 0.88 (0.01) | 0.89 (0.01) |
|  |  | Diffuse & AlignedSV | 0.91 (0.02) | 0.99 (0.01) |  | 0.94 (0.01) | 0.99 (0.01) |  | 0.90 (0.01) | 0.97 (0.01) |
|  |  |  |  |  |  |  |  |  |  |  |
|  | 500 | DivergentSV | 0.91 (0.01) | 0.97 (0.03) |  | 0.93 (0.01) | 0.94 (0.01) |  | 0.91 (0.01) | 0.93 (0.02) |
|  |  | Diffuse & AlignedSV | 0.91 (0.01) | 1.00 (0.00) |  | 0.94 (0.01) | 1.00 (0.00) |  | 0.92 (0.01) | 1.00 (0.00) |
| **Population PPP** | |  |  |  |  |  |  |  |  |  |
|  | 200 | DivergentSV | 0.00 (0.00) | 0.00 (0.03) |  | 0.00 (0.00) | 0.00 (0.00) |  | 0.00 (0.00) | 0.00 (0.00) |
|  |  | Diffuse & AlignedSV | 0.00 (0.00) | 0.52 (0.26) |  | 0.00 (0.00) | 0.38 (0.03) |  | 0.00 (0.00) | 0.52 (0.27) |
|  |  |  |  |  |  |  |  |  |  |  |
|  | 500 | DivergentSV | 0.00 (0.00) | 0.05 (0.09) |  | 0.00 (0.00) | 0.00 (0.00) |  | 0.00 (0.00) | 0.01 (0.04) |
|  |  | Diffuse & AlignedSV | 0.00 (0.00) | 0.51 (0.26) |  | 0.00 (0.00) | 0.49 (0.25) |  | 0.00 (0.00) | 0.50 (0.28) |
| Note:  C1-1= F3-*I*10, crt3; C1-3 = F3-*I*10, mis1; C2-1 = F6-*I*5, crt6; C2-3 = F6-*I*5, mis2; C3-1 = F6-*I*10, crt6; C3-3 = F6-*I*10, mis2;  For model name, F = # of latent factors, *I* = # items, crt = correct specification, mis=misspecification. For example, “F3-*I*5*, crt3*” reads as: three-factor model with five items correctly specified as a three-factor model., and “F3-*I*5*, mis2*” reads: three-factor model with five items misspecified as a two-factor model. | | | | | | | | | | |
|  | | | | | | | | | | |

| **Table A5**  *Study 2: Mean (SD) of BRMSEA, BCFI, and BTLI and PPp by sample size, prior specification, cross-loading magnitude, and cross-loading misspecification.* | | | | | | | | | | |
| --- | --- | --- | --- | --- | --- | --- | --- | --- | --- | --- |
|  |  |  | **Three factors:**  **Mis-specified as one**  **and Correct** | | **Six factors:**  **Mis-specified as two**  **and Correct** | | |  | **Six factors:**  **Mis-specified as two**  **and Correct** | |
|  |  | **Models** | **D1-3** | **D1-1** |  | **D2-3** | **D2-1** |  | **D3-3** | **D3-1** |
|  | **n** | **Prior Type** | **F3-I10-cld6, 0** | **F3-I10-cld6, 6** |  | **F6-I5-cld6, 0** | **F6-I5-cld6, 6** |  | **F6-I10-cld12, 0** | **F6-I10-cld12, 12** |
| **Population BRMSEA** | |  | **0.041** | **0.000** |  | **0.034** | **0.000** |  | **0.028** | **0.000** |
|  | 200 | DivergentSV | 0.07 (0.00) | 0.07 (0.01) |  | 0.07 (0.01) | 0.07 (0.00) |  | 0.06 (0.00) | 0.06 (0.01) |
|  |  | Diffuse & AlignedSV | 0.06 (0.01) | 0.02 (0.01) |  | 0.07 (0.01) | 0.02 (0.01) |  | 0.05 (0.01) | 0.03 (0.00) |
|  |  |  |  |  |  |  |  |  |  |  |
|  | 500 | DivergentSV | 0.07 (0.00) | 0.02 (0.01) |  | 0.07 (0.00) | 0.07 (0.00) |  | 0.06 (0.00) | 0.02 (0.01) |
|  |  | Diffuse & AlignedSV | 0.04 (0.00) | 0.01 (0.01) |  | 0.06 (0.01) | 0.01 (0.01) |  | 0.03 (0.00) | 0.01 (0.00) |
| **Population BCFI** | |  | **0.976** | **1.000** |  | **0.982** | **1.000** |  | **0.978** | **1.000** |
|  | 200 | DivergentSV | 0.92 (0.01) | 0.93 (0.02) |  | 0.92 (0.01) | 0.93 (0.01) |  | 0.88 (0.01) | 0.90 (0.02) |
|  |  | Diffuse & AlignedSV | 0.95 (0.01) | 1.00 (0.00) |  | 0.93 (0.01) | 0.99 (0.01) |  | 0.92 (0.01) | 0.98 (0.01) |
|  |  |  |  |  |  |  |  |  |  |  |
|  | 500 | DivergentSV | 0.93 (0.01) | 0.99 (0.00) |  | 0.93 (0.01) | 0.93 (0.01) |  | 0.90 (0.01) | 0.98 (0.01) |
|  |  | Diffuse & AlignedSV | 0.97 (0.00) | 1.00 (0.00) |  | 0.95 (0.01) | 1.00 (0.00) |  | 0.97 (0.01) | 1.00 (0.00) |
| **Population BTLI** | |  | **0.974** | **1.000** |  | **0.980** | **1.000** |  | **0.977** | **1.000** |
|  | 200 | DivergentSV | 0.91 (0.01) | 0.93 (0.02) |  | 0.91 (0.01) | 0.92 (0.01) |  | 0.88 (0.01) | 0.90 (0.02) |
|  |  | Diffuse & AlignedSV | 0.95 (0.02) | 0.99 (0.00) |  | 0.93 (0.01) | 0.99 (0.01) |  | 0.92 (0.01) | 0.98 (0.01) |
|  |  |  |  |  |  |  |  |  |  |  |
|  | 500 | DivergentSV | 0.93 (0.01) | 0.99 (0.00) |  | 0.92 (0.01) | 0.93 (0.01) |  | 0.90 (0.01) | 0.98 (0.01) |
|  |  | Diffuse & AlignedSV | 0.97 (0.00) | 1.00 (0.00) |  | 0.94 (0.01) | 1.00 (0.00) |  | 0.96 (0.01) | 1.00 (0.00) |
| **Population PPP** | |  |  |  |  |  |  |  |  |  |
|  | 200 | DivergentSV | 0.00 (0.00) | 0.01 (0.06) |  | 0.00 (0.00) | 0.00 (0.00) |  | 0.00 (0.00) | 0.00 (0.00) |
|  |  | Diffuse & AlignedSV | 0.00 (0.01) | 0.55 (0.25) |  | 0.00 (0.00) | 0.39 (0.29) |  | 0.00 (0.00) | 0.51 (0.28) |
|  |  |  |  |  |  |  |  |  |  |  |
|  | 500 | DivergentSV | 0.00 (0.00) | 0.12 (0.13) |  | 0.00 (0.00) | 0.00 (0.00) |  | 0.00 (0.00) | 0.05 (0.10) |
|  |  | Diffuse & AlignedSV | 0.00 (0.00) | 0.51 (0.25) |  | 0.00 (0.00) | 0.51 (0.24) |  | 0.00 (0.00) | 0.50 (0.28) |
| Note:  D1-1= F3-*I*10-cld6,6; D1-3 = F3-*I*10-cld6,0; D2-1 = F6-*I*5-cld6,6; D2-3 = F6-*I*5-cld6,0; D3-1 = F6-*I*10-cld12,12;  D3-3 = F6-*I*10-cld12,0  For model name, F = # of latent factors, *I* = # items, and cld =# of cross-loading. For example, “F6-*I*10-cld12, 12” should be read as: Six-factor model with ten items and 12 cross-loadings specified as having 12 cross-loadings. | | | | | | | | | | |

| **Table A6**  *Study1: Mean (SD) of PPP by sample size, prior specification, covariance structure and model factor misspecification.* | | | | | | | | | | |
| --- | --- | --- | --- | --- | --- | --- | --- | --- | --- | --- |
|  |  |  | **Three factors mis-specified as one (Model A3)** | | **Three factors mis-specified**  **as two (Model A2)** | | |  | **Three factors correctly specified (Model A1)** | |
|  | **n** | **Prior Type** | **A31_cor.35** | **A31_cor.85** |  | **A32_cor.35** | **A32_cor.85** |  | **A33_cor.35** | **A33_cor.85** |
| *PPP* | 75 | DivergentSV | 0.00 (0.00) | **0.07 (0.12)** |  | 0.00 (0.00) | **0.08 (0.12)** |  | **0.13 (0.18)** | **0.09 (0.13)** |
|  |  | Other four priors | 0.00 (0.00) | **0.11 (0.15)** |  | 0.00 (0.00) | **0.23 (0.20)** |  | **0.54 (0.24)** | **0.41 (0.27)** |
|  | 200 | DivergentSV | 0.00 (0.00) | 0.00 (0.01) |  | 0.00 (0.00) | 0.00 (0.00) |  | **0.22 (0.16)** | 0.00 (0.01) |
|  |  | Other four priors | 0.00 (0.00) | 0.00 (0.01) |  | 0.00 (0.00) | 0.04 (0.09) |  | **0.55 (0.23)** | **0.46 (0.26)** |
|  |  |  |  |  |  |  |  |  |  |  |
|  | 500 | DivergentSV | 0.00 (0.00) | 0.00 (0.00) |  | 0.00 (0.00) | 0.00 (0.00) |  | **0.27 (0.18)** | 0.00 (0.00) |
|  |  | Other four priors | 0.00 (0.00) | 0.00 (0.00) |  | 0.00 (0.00) | 0.00 (0.00) |  | **0.53 (0.24)** | **0.52 (0.23**) |
|  |  |  |  |  |  |  |  |  |  |  |
|  | 2000 | DivergentSV | 0.00 (0.00) | 0.00 (0.00) |  | 0.00 (0.00) | 0.00 (0.00) |  | **0.40 (0.22)** | **0.23 (0.15)** |
|  |  | Other four priors | 0.00 (0.00) | 0.00 (0.00) |  | 0.00 (0.00) | 0.00 (0.00) |  | **0.51 (024)** | **0.48 (0.23)** |
|  | |  |  |  |  |  |  |  |  |  |
| *Note: PPPPs are generated for only small variance plus in Mplus (Version. 8.7, 2021)*  A33_cor.85=Three factor model, not mis-specified; A32_cor.85=Three factor model, mis-specified as two factors. A31_cor.85 =Three factor model, mis-specified as one factor; A33_cor.35=Three factor model, not mis-specified A32_cor.35=Three factor model, mis-specified as two factors; A31_cor.35=Three factor model, mis-specified as one factor.  AllignedLV=Aligned with large variance; AllignedSV=Aligned with small variance; DivergentLV=Divergent with large variance; DivergentSV=Divergent with small variance  Other four priors: AllignedLV, AllignedSV, DivergentLV, & Diffuse prior | | | | | | | | | | |

| **Table A7**  *Study 1: Mean (SD) of PPP by sample size, prior specification, cross-loading magnitude, and cross-loading misspecification.* | | | | | | | | | | |
| --- | --- | --- | --- | --- | --- | --- | --- | --- | --- | --- |
|  |  |  | **Three cross-loadings with all three mis-specified (Model B1)** | | **Three cross-loadings mis-specified as two (Model B2)** | | |  | **Three cross-loadings correctly specified (Model B3)** | |
|  | **n** | **Prior Type** | **B30_cld.2** | **B30_cld.5** |  | **B32_cld.2** | **B32_cld.5** |  | **B33_cld.2** | **B33_cld.5** |
| *PPP* | 75 | DivergentSV | 0.00 (0.04) | 0.00 (0.00) |  | 0.01 (0.04) | 0.00 (0.01) |  | 0.01 (0.07) | 0.05 (0.12) |
|  |  | Other four priors | **0.34 (0.24)** | 0.00 (0.01) |  | **0.46 (0.25)** | 0.10 (0.13) |  | **0.53 (0.25)** | **0.51 (0.25)** |
|  | 200 | DivergentSV | 0.02 (0.04) | 0.00 (0.00) |  | 0.10 (0.11) | 0.00 (0.00) |  | 0.19 (0.15) | 0.22 (0.16) |
|  |  | Other four priors | **0.15 (0.15)** | 0.00 (0.00) |  | **0.40 (0.22)** | 0.00 (0.01) |  | **0.55 (0.22)** | **0.53 (0.22)** |
|  |  |  |  |  |  |  |  |  |  |  |
|  | 500 | DivergentSV | 0.00 (0.01) | 0.00 (0.00) |  | 0.05 (0.08) | 0.00 (0.00) |  | 0.24 (0.16) | **0.25 (0.16)** |
|  |  | Other four priors | 0.01 (0.03) | 0.00 (0.00) |  | **0.18 (0.17)** | 0.00 (0.00) |  | **0.52 (0.22)** | **0.50 (0.22)** |
|  |  |  |  |  |  |  |  |  |  |  |
|  | 2000 | DivergentSV | 0.00 (0.00) | 0.00 (0.00) |  | 0.00 (0.01) | 0.00 (0.00) |  | 0.37 (0.20) | **0.41 (0.21)** |
|  |  | Other four priors | 0.00 (0.00) | 0.00 (0.00) |  | 0.00 (0.01) | 0.00 (0.00) |  | **0.49 (0.22)** | **0.52 (0.22)** |
|  | |  |  |  |  |  |  |  |  |  |
| *Note: PPPPs are generated for only small variance plus in Mplus (Version. 8.7, 2021)*  B33_cld.2=three Cross-loadings, non-mis-specified; B32_cld.2=three Cross-loadings, one mis-specified B30_cld.2=three Cross-loading, all three-mis-specified; B33_cld.5=three Cross-loadings, non-mis-specified B32_cld.5=three Cross-loadings, one mis-specified; B30_cld.5=three Cross-loading, all three-mis-specified  AllignedLV=Aligned with large variance; AllignedSV=Aligned with small variance; DivergentLV=Divergent with large variance; DivergentSV=Divergent with small variance  Other four priors: AllignedLV, AllignedSV, DivergentLV, & Diffuse prior | | | | | | | | | | |

| **Table A8**  Overview of literature on Bayesian fit measures | | | | |
| --- | --- | --- | --- | --- |
| Studies | Model | Priors | Fit Measures | Major Findings |
| Hoofs, H., van de Schoot, R., Jansen, N. W. H., & Kant, Ij. (2018). | CFA (1 or 2-factor models, with 6 to 12 indicators) | Three prior variations:  a). M*plus* default prior.  b).  *N*($\mu=correct parameter values$, $\sigma^{2}=0.05)$for intercepts and factor loadings  c). *N*($\mu=wrong parameter values$, $\sigma^{2}=0.005)$ for intercepts and factor loadings | BRMSEA | The BRMSEA functions similarly to the RMSEA in large samples.  The 90% posterior probability interval of the BRMSEA is valid for evaluating model fit in large samples (N = 1,000), using cutoff values for the lower limit (.05) and upper limit (.08) as a guideline. |
| Garnier-Villarreal, M., and Jorgensen, T. D. (2020). | CFA & SEM (3 or 4-factor models, with 6 to 12 indicators) | M*plus* default Non-informative priors | BRMSEA^DevM^, BCFI, BTLI, BMc, BNFI, B(Gamma-Hat), and B(adj Gamma-Hat) | The posterior means of these Bayesian fit indices were similar to their frequentist counterparts when estimated with noninformative priors.  BRMSEA^DevM^ formulation more closely approximates the frequentist RMSEA than the Hoofs et al. (2018) BRMSEA formulation. |
| Liang (2020). | CFA (2-factor models, with 5 indicators for each factor) | Seven shrinkage prior choices on cross-loadings: *N*($\mu=0$, $\sigma^{2})$. With variances ranging from 0.005 to 0.08  Five Inverse Gamma and Normal noninformative/vague priors on intercepts, factor loadings, and error variances. | BRMSEA^DevM^, BCFI, BTLI (also PPP, PPPP, BIC and DIC). | BCFI, BTLI, and BRMSEA were insensitive to choices of both noninformative/vague priors and shrinkage priors. |
| Winter, S.D., & Depaoli, S. (2022b). | 5-timepoint LGM with a linear and quadratic slope | Three prior variations:   1. M*plus* default prior. 2. Prior with mean aligned with population value 3. Prior with mean divergent from population value   For (b) and (c), SD = 0.3 for the intercept mean and 0.1 for the slope mean | BRMSEA^DevM^, BCFI, BTLI, PPP-value | BRMSEA, BCFI, BTLI may be more useful for model selection than for assessing the fit of a single model.  As sample size increased, BRMSEA, BCFI, BTLI were more likely to indicate good fit for misspecified models.  Diverging priors can make correctly specified models appear misspecified, especially with smaller sample sizes (n=50 and 100). Led to inflated model rejection rates (especially for BRMSEA).  BRMSEA performed better than the BCFI and BTLI in assessing misspecification in the marginal mean structure.  PPP-value seems to have performed better overall for n>50 but $\leq500$; it was however unlikely to identify severe misspecifications for n $\leq$ 50. |
| Edwards, K. D., & Konold, T. R., (2022). | CFA (2-factor model with 12 indicator) | Primary Factor Loadings: Nine different Bayesian prior: One diffuse prior [*N*($0,{10}^{10}$)] and eight informative priors with varying levels of accuracy and informativeness.  Cross-Loadings: Two different prior specifications: *N*($0, 0.01$) and *N*($0, 0.05$)  All other model parameters: M*plus* defaults | BRMSEA^DevM^, BCFI, BTLI, PPP-value | Inaccurate and informative priors resulted in biased fit indices estimates, especially with small sample sizes.  Coverage rates for the 90% credibility intervals were below the nominal 90% for the strongly informative inaccurate prior, even when the model was correctly specified.  Generally, BRMSEA^DevM^, BCFI, BTLI were impacted by prior choice, particularly when sample sizes are small. |
| Cao, C., Lugu, B., & Li, J. (2023). | SEM | Three prior variations:   1. M*plus* default prior. 2. Prior with mean aligned with population value 3. Prior with mean divergent from population value   For (b) mean = 0.4 or 0.8, and For (c) mean=0.6. For both (b) and (c), Variance ranges from 0.005 to 0.04 | BRMSEA^DevM^, BCFI, BTLI, PPP-value, and DIC | BRMSEA^DevM^, BCFI, and BTLI was not sensitive to structural misspecification.  Informative priors with narrow precision led to high false positive rates.  PPP and DIC effectively detected misspecification under optimal conditions (e.g., large sample size, n=500, and high factor loadings, 0.80). |
| Konold, T.R. & Sanders, E.A. (2023). | CFA (3-factor, 9 to 15 indicator model | For factor loadings, the following priors were specified: Non-informative prior, *N*($0,{10}^{10}$); Weakly informative prior, *N*($0.60, 0.04$); Strongly informative prior, *N*($true parameter values$, $0.01)$. For correlations: *U*(-1,1); For indicator residual variance priors: *IG*(3,1); and Mplus’ default non-informative priors for all others. | RMSEA, BRMSEA, CFI, BCFI, TLI, BTLI, PPP, Gamma-hat, Adjusted Gamma-hat | The BRMSEA, Gamma-hat, and Adjusted Gamma-hat, are susceptible to the reliability paradox just like the RMSEA, showing worse fit for models with better measurement quality. BCFI and BTLI, like the CFI and TLI, do not exhibit the reliability paradox, showing better fit as measurement quality increases. The PPP performed better in identifying structural misspecifications, and was less affected by reliability paradox. |

**Note:** M*plus* non-informative default priors are defined and assigned as follow: intercepts and factor loadings *t* ~ *N*(0, $\infty$), residual variances *d* ~ *IG*(-1, 0), and latent factor covariances *F* ~ *IW*(0, -7), (M*plus* 8.7, Muthén and Muthén, 1998 – 2021).

**Appendix: Figures**

**Figure A1**

*Effect of prior specification, sample size and model latent factor misspecification on PPP*


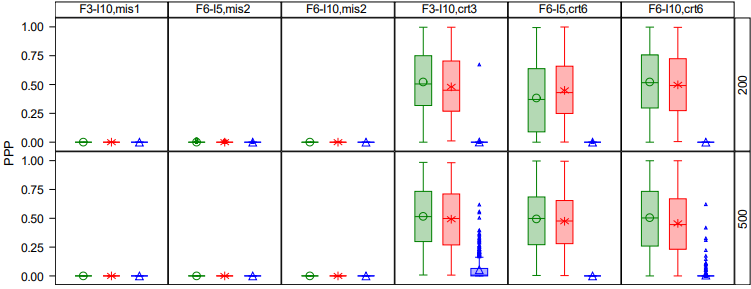


Model C1-3

Model C2-3

Model C3-3

Model C1-1

Model C2-1

Model C3-3


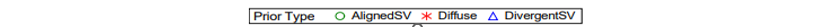


Note:

C1-1= F3-*I*10, crt3; C1-3 = F3-*I*10, mis1; C2-1 = F6-*I*5, crt6; C2-3 = F6-*I*5, mis2; C3-1 = F6-*I*10, crt6; C3-3 = F6-*I*10, mis2;

For model name, F = # of latent factors, *I* = # items, crt = correct specification, mis=misspecification. For example, “F3-*I*5*, crt3*” reads as: three-factor model with five items correctly specified as a three-factor model., and “F3-*I*5*, mis2*” reads: three-factor model with five items misspecified as a two-factor model.

**Figure A2**

*Effect of prior specification, sample size and model cross-loading misspecification on PPP*


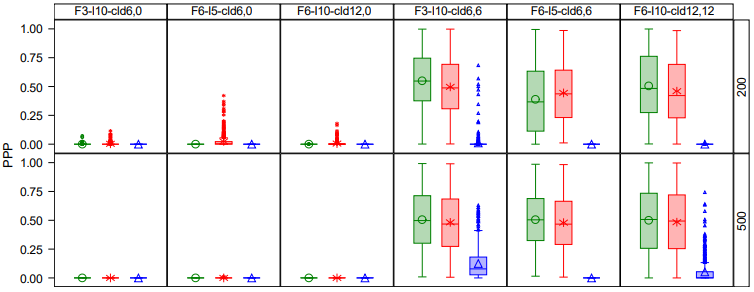


Model D1-3

Model D2-3

Model D3-3

Model D1-1

Model D2-1

Model D3-3


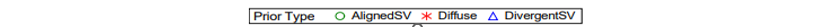


Note:

D1-1= F3-*I*10-cld6,6; D1-3 = F3-*I*10-cld6,0; D2-1 = F6-*I*5-cld6,6; D2-3 = F6-*I*5-cld6,0; D3-1 = F6-*I*10-cld12,12;

D3-3 = F6-*I*10-cld12,0

For model name, F = # of latent factors, *I* = # items, and cld =# of cross-loading. For example, “F6-*I*10-cld12, 12” should be read as: Six-factor model with ten items and 12 cross-loadings specified as having 12 cross-loadings.

**Figure A3**

*Scatterplots (upper layer) of two different performances with PPP-values both close to 0.5 and the corresponding comparisons (lower layer) of probability densities function. Reproduced from*

*Wu, Yuen, & Leung, (2014).*


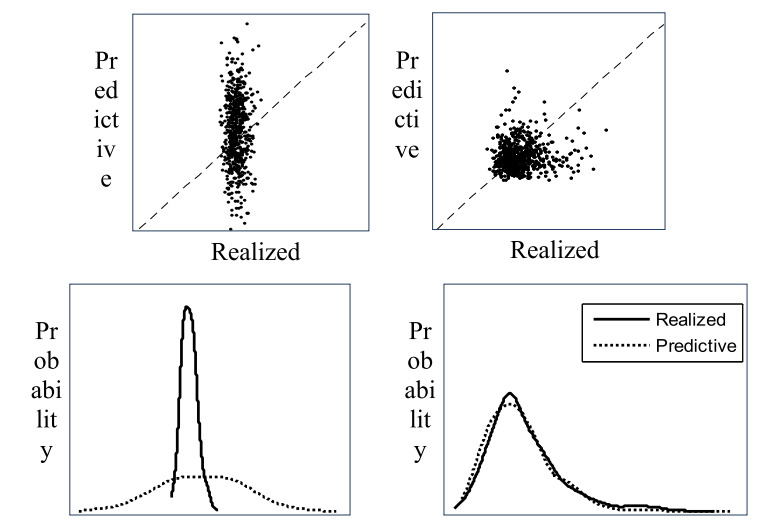


*Note*. By numerical assessment only (PP*p* close to 0.5), the left and right models would have been adjudged equally fitting.
